# Investigation of outcome measures and anomalous lower extremity in osteoarthritis patients with Jumpstart nutrition® supplementation

**DOI:** 10.22088/cjim.15.1.1

**Published:** 2024

**Authors:** Ganguly Apurba

**Affiliations:** 1 Department of Biochemistry, Techno India University, Salt Lake, Kolkata, India

**Keywords:** Osteoarthrosis, Dietary supplement, Abnormal lower extremity, Aberrant outcome measures, Symptomatic slow-acting drugs.

## Abstract

**Background:**

Osteoarthritis (OA) is characterized by cartilage and synovial inflammation as well as anomalous lower extremity leading to joint pain, and impairment in lifestyle and epidemic of obesity. This study aimed to use the Jumpstart Nutrition® supplement (JNS) for achieving symmetry of aberrant lower extremity and improving the outcome measures in the management of OA.

**Methods:**

This week-twelve registry included 108 patients treated with JNS mainly comprised of calcium, phosphorus, magnesium, vitamin-K_2_, coenzyme-Q_10_, vitamin-C, boswellic acids, and curcumin mixed with soy and whey proteins (experimental group) and 72 were treated with symptomatic slow-acting drugs (control group) for chronic OA confirmed with radiological images. The outcome measures (Visual analogue scale, Western Ontario and McMaster Universities Osteoarthritis Index, Knee-injury Osteoarthritis Outcomes Scale, and Body mass index), and anomalous lower extremity included bilateral: knee gaps between biceps femoris-short head and surface of the bed, diameters of muscles at the calf, the thigh, 4cm above and below the patella, angles of straight leg raising, knee- flexion and-extension in supine were evaluated with appropriate protocol at week-0 and at week-12 for both the groups.

**Results:**

After week-12, risk ratios of studied lower extremity, and mean ±standard deviation of all outcome measures were significantly improved (p<0.0001), and Kellgren-Lawrence scale (KLS) was upgraded to ≥2 in experimental group compared to control.

**Conclusions:**

This registry study indicates that JNS can be used to achieve symmetry of studied lower extremity and to improve the outcome measures safely as an effective management of OA patients confirmed with radiological images correlated with KLS.

Osteoarthrosis (OA) is a major cause of disability and impaired quality of life, with a significant impact on healthcare costs (1-2). World Health Organization has reported that nearly 130 million people will suffer from OA worldwide by 2050 and the women will be almost double of the men over the age of 60 years, constituting a significant social burden (3-4). The principal objectives of the treatment of OA are to relive pain, to sustain joint flexibility, to optimize joint and limb functions and to enhance quality of life. Muscle relaxants may occasionally relieve pain (although they can cause significant side effects in the elderly). Oral corticosteroids have generally no role in OA, although intra-articular depot corticosteroids can relieve pain and increase joint flexibility in selected patients (2). Synthetic hyaluronans (comparable to hyaluronic acid) can be injected in the knee joint, obtaining pain relief for prolonged periods of time but the effects on the evolution of the disease are limited. 

Therefore, this new evidence could potentially increase public interest in the benefits of alternative treatments. Currently 69% of patients with OA take some form of dietary supplements for their condition as an alternative therapy (5) The first choice of supplementary treatment is commonly recommended with symptomatic slow-acting drugs for osteoarthritis (SYSADOA) which include glucosamine sulfate, glucosamine hydrochloride, chondroitin sulfate, hyaluronic acid, diclofenac sodium 2%, avocado soybean unsaponifiables (ASU) (1:2 avocado and soybean oils), and diacerein to be used two times a day (5-8) to control pain and flexibility of muscles and to control erosion of bones temporarily, but all have tremendous side-effects in the long run (8-13). Non-steroidal anti-inflammatory drugs (NSAIDs) including cyclooxygenase-2 (COX-2) inhibitors, either do not modify the disease course or have been associated with serious renal and cardiovascular effects and gastrointestinal bleeding (14). At the same time, the clinical guidelines of these dietary supplements are still controversial, and their clinical benefits or herms have not been established. 

In this study, Jumpstart Nutrition® supplement (JNS) is suggested as the most effective with low cost alternative in the management of OA (15) The JNS is composed of calcium, phosphorus, magnesium, and iron as minerals required for improving bones and muscles health, coenzyme- Q_10_ (ubiquinone), vitamin-K_2 _(menaquinone), vitamin-C, folic acid, and vitamin-D_2 _as essential vitamins required for muscles and bones, boswellic acids and curcumin as antioxidants for relieving pain, inflammation, and stiffness of muscles and natural protein powers of soy and whey for promoting mainly bone growth and muscle strength, according to their dietary reference intakes as recommended by the Food and Nutrition Board of the Institute of Medicine, National Academy of Sciences (Washington, DC, USA) (16-18) to treat OA patients. Compared to the conventional supplementary treatment with SYSADOA (5-8).

Furthermore, it is to be noted that nano curcumin (19) and boswellic acids (20) contained in JNS have proven to inhibit activation of nuclear factor kappa B (NF-kB) (21-22) a potent inducer of chronic inflammation and pain (23) whereas, calcium, phosphorus, vitamin-K_2_, coenzyme-Q_10_, vitamin C, folic acid contained in JNS have proven to inhibit the strength of the muscles, thereby the gap between the tibiofemoral and patella-femoral joints increased because of coenzyme Q_10 _(24-26) and at the same time, osteoblasts (cells that build up) produce inactive osteocalcin and it needs vitamin K_2_ to become fully activated and bind calcium to make the skeleton stronger and less susceptible to fracture (27) Again, the matrix Gla protein (MGP) of vitamin- K_2_ is a central calcification inhibitor produced by the cells of vascular smooth muscles and regulates the potentially fatal accumulation of calcium which keeps calcium from accumulating in the walls of blood vessels (28).

The aim of this present supplement registry study was to conduct a pilot assessment of a new preparation (JNS) in subjects with OA, to maintain the symmetry in the lower extremity correlated with radiographic changes evaluated by Kellgren-Lawrence grading scale (29) and substantial improvement of pain and functional activities evaluated by visual analogue scale (VAS) (30) The Western Ontario and McMaster Universities Osteoarthritis Index (WOMAC) (31) Knee-injury and osteoarthritis outcome score (KOOS) (32) and Body mass index (BMI) (33) cost-effectively in comparison with OA subjects treated with SYSADOA (5-8). 

## Methods

**Recruitment of patients**: Out of 369 approached cohorts, a total of 334 Indian patients of different ethnics group, aged 45 to 75 years old, suffering for more than six years with OA who were treated at OPTM Health Care (P) Ltd (License Number 34218956 under The West Bengal Clinical Establishment Act 1950, India) from November 2018 to January 2019. Were included in this 12-week registry. OPTM Research Institute Ethics Committee has evaluated and approved the present study protocol. An institutional review board-approved consent form for the physical examination, and radiological images required for the study was signed by all patients in the first phase of the screening procedure*. *The eligibility criteria for this study were age between 45-75 years, clinical knee OA confirmed by radiography (osteophytes and/or joint space narrowing assed by radiologist), continues ongoing or recurrent chronic pain more than three to six months, and a BMI>30 kg/m^2^.

**Exclusion criteria: **One hundred fifty-four of 334 patients with concomitant diseases or risk conditions requiring drug treatment, severe metabolic disorders, drugs/alcohol addiction and /or psychiatric diseases, oncological conditions, pregnant, planned conception, multiple drug dependence, a history of cancer including caranomatosis and granulocytic leukemia, a history of chronic liver, kidney, and heart diseases, patients with amputated legs and also patients who did not agree to a physical and radiological evaluations, and /or attend weekly follow-up visits were all included in exclusion criteria.

**Figure 1:**
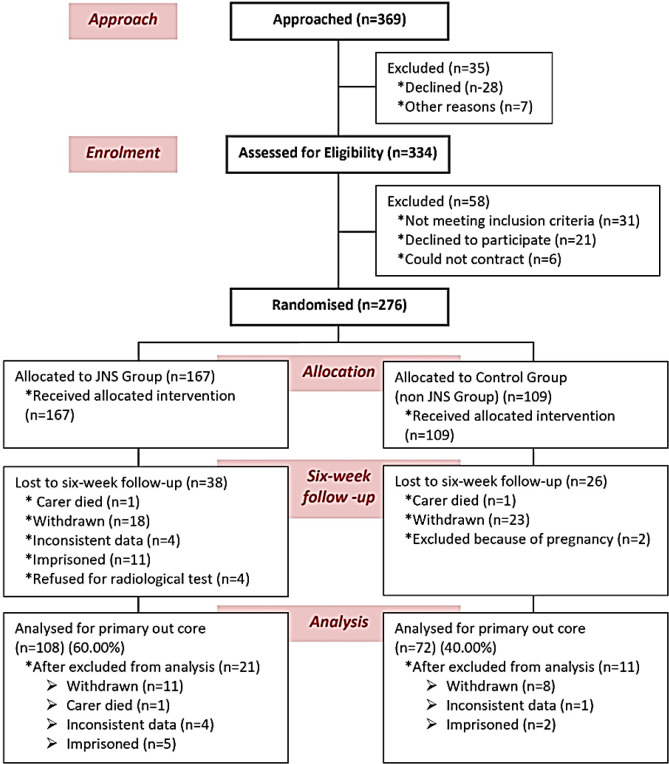
Study flow chart according to Consolidated Standards of Reporting Trials (CONSORT)

**Figure 2:**
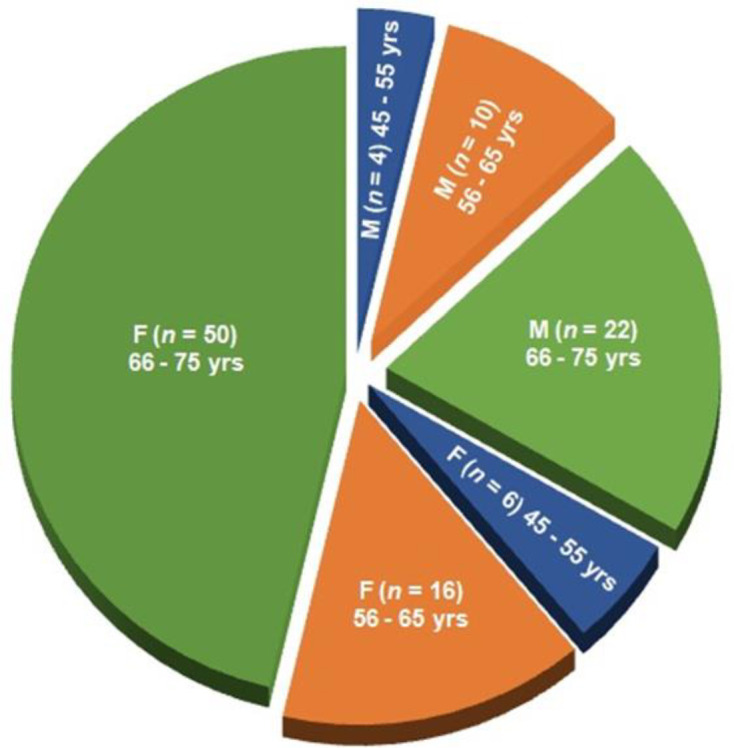
Classification of age groups (45-55, 56-65 and 66-75 years) of 108 experimental patients, 36male-only and 72female-only using Jumpstart Nutrition® supplement

**Figure 3:**
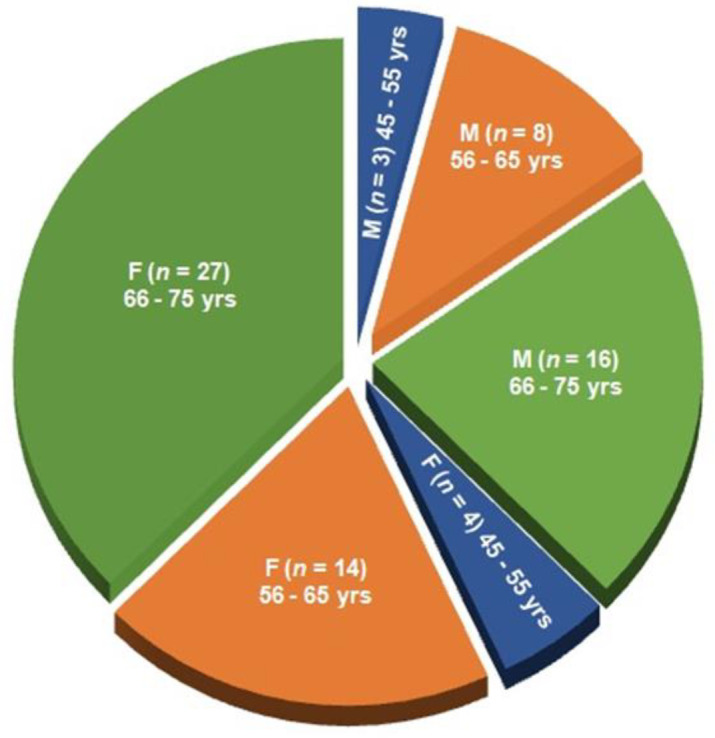
Classification of age groups (45-55, 56-65 and 66-75 years) of 72 control patients, 27 male-only and 45 female-only without using Jumpstart Nutrition® supplement

**Study design: **After evaluating the exclusion criteria, 108 of the remaining 180 patients suffering with acute OA confirmed with radiological images having severe pain with disable lifestyle because of abnormal lower anatomical features were treated with JNS considered as experimental patients and the remaining 72 subjects were treated with with symptomatic slow-acting drugs for osteoarthritis (SYSADOA) which include glucosamine sulfate, glucosamine hydrochloride, chondroitin sulfate, hyaluronic acid,diclofenac sodium 2%*,* avocado soybean unsaponifiables (ASU) (1:2 avocado and soybean oils), and diacerein considered as control subjects. The study flow chart according to consolidated standards of reporting trials is shown in figure 1. The gender-wise classifications of age groups 45-55, 56-65, and 66-75 years of the subjects for both the groups are shown in figures 2-3. 

**Evaluation of anatomical features** *: *Physical examinations were evaluated at week 0 at week 12 of post-treatment including anatomical measurements such as bilateral gap at the knees between the point of short head of the biceps femoris at the lateral knee and the surface of the bed while supine (KGB), bilateral diameter of muscles at the thighs (DTM), bilateral diameter of muscles of the calves (DCM), bilateral diameter of muscles connected with the knee joints 4 cm above the patella (DAP) and 4 cm below the patella (DBP), bilateral straight legs raising in supine (SLR), bilateral angles of flexion insupine (KFS), and bilateral angles of extension in supine (KES).

A meter scale was used to measure KGB. The parameters of DTM, DCM, DAP and DBP were measured using a meter tape and a goniometer was used for straight leg raising, flexion and extension measurements based on the American Academy of Orthopedic Surgeons (AAOS) ,1965 (34). The mean, standard deviation (SD), and their mean differences (MDs), 95% confidence intervals (CIs), and *p*-values, risk ratios, correlation coefficient and their *p*-values were evaluated at week 0 and at week 12 for both the groups according to their age groups of 45-55, 56-65; and 66-75 years. 

**Evaluation of pain under visual analogue scale**: Visual analogue scale (VAS) for pain is a one-dimensional measure of pain intensity[30] Observation of patient’s perceived symptoms of pain intensity in the last 24 hours was point out on the line of 100 mm. The pain intensity marked as no pain (0-4 mm), mild pain (5-44 mm), moderate pain (45-74 mm), and severe pain (75-100 mm) separately for right leg, left leg, and lower back pain under the scale was evaluated for each patient at the baseline and at the end of 12-week for both the groups*. *Their mean, SD, and p-values, and the mean percentage of improvement and declination were evaluated at the end of 12-week for all the patents separately*.*

**Evaluation of pain, stiffness and physical function under the Western Ontario and McMaster Universities Osteoarthritis Index: **The scoring data for pain, stiffness and functional disability of individual patient were evaluated by using the Western Ontario and McMaster Universities Osteoarthritis Index (WOMAC) as per method followed by previous researcher (31) The mean, SD and p-values and the improvementand declination of pain, stiffness and physical functional abilities were evaluated at week 12 for all the patents for both the groups separately.

**Evaluation of Knee-injury Osteoarthritis Outcomes Scale: **The Knee-injury Osteoarthritis Outcomes Scale (KOOS) developed by Ewa Roos and co-authors in the 1990s to assess the patient’s opinion about their knee and associated problems including quality of life as an instrument (32) A Likert scale is used and all items have five possible answer options scored from 0 to 4 and each of the five scores is calculated as the sum of the items included. It holds 42 items in five separately scored subscales experienced during last week such as pain (0-36); other symptoms (0-28); function, daily living or activities of daily living (ADL) (0-68); function, sport and recreation activities (0-20); and quality of life (QOL) (0-16). Scores are transformed to a 0-100 scale with zero representing extreme knee problems and 100 representing no knee problems as common in orthopedic assessment scales and generic measures. Scores between 0 and 100 represent the percentage of total possible score achieved. All the scoring data under KOOS were evaluated separately for each cohort of experimental and control groups at week 0 and week 12. Their mean, SD and p-values for both overall and separately by gender were also graphically evaluated. The knee-injury osteoarthritis outcomes scale (KOOS) was developed by Ewa M. Roos and co-authors in the 1990s to assess the patient’s opinion about their knee and associated problems as an instrument (32). 

**Evaluation of Body mass index:**Body weight in kilogram was measured without shoes or heavy clothing using an electronic scale. Height in meter was measured without shoes using a wall-mounted stadiometer (33) Body mass index (BMI, kg/m^2^) was calculated for all the patients based on measured weights and heights at pre- and post-treatment for both the groups. Their mean, SD and p-values and the improvement and declination were evaluated at week 12 for all the patents separately

**Evaluation of radiological images with the Kellgren-Lawrence grading scale**: The Kellgren and Lawrence system is a common method of classifying the severity of knee osteoarthritis (OA) developed by Kellgren et al. (29) The KL classification was originally described using anterior-posterior (AP) knee radiographs based on the five grades such as Grade 0, which demonstrates no radiographic features of OA present; Grade 1, which demonstrates doubtful joint space narrowing (JSN) with possible osteophyte lipping; Grate 2, which demonstrates possible JSN with definite osteophyte formation; Grade 3, which demonstrates definite JSN, moderate osteophyte formation, some sclerosis and possible deformity of bony ends and Grade 4, which demonstrates large osteophyte formation, severe JSN with marked sclerosis and definite deformity of bony ends. Radiological images for both knee joints of 180 patients were collected, both anterior-posterior (AP) and lateral views, at week 0 and week 12. The AP views of the knee joints of 180 patients were assessed by K-L grading scale. The AP view of knee joints x-ray images of two such patients, (before and after the treatment) are separately evaluated for both the groups. 

**Management of supplement studies: **The formulation of the JNS has been scientifically evaluated by Nanophyto wellness Pvt Ltd, Kolkata, India. As discussed in the previous study (15) the pharmacokinetics of the ingredients used in the supplement along with their dietary reference intakes (DRIs) as recommended by the Food and Nutrition Board of the Institute of Medicine, National Academy of Sciences (USA) (16) has been strictly scrutinized by the Food Safety and Standards Authority of India (FSSAI) before issuing the license (bearing the number 10018031002579) to the company for distribution of the products (17-18).

It is composed of 747 mg of minerals (calcium, phosphorus, magnesium, iron in the ratio 5:4: 2:0.21), 125.13 mg of vitamins (coenzyme-Q_10_, vitamin-C, folic acid, vitamin-K_2_, and vitamin-D_2 _in the ratio 100mg: 25mg: 100µg: 20µg: 8µg), and 275 mg of phytonutrients (boswellic acids and curcumin in the ratio 8:3) mixed with soya and whey proteins in the ratio 2:1 to make a dose of 25 g per serving with 250 ml of cold water. The minerals were collected from Mitushi Biopharma, Ahmedabad, Gujarat, India, vitamins were collected from Herbo Nutra, Razapur Khurd, New Delhi and other phytonutrients were collected from Sami Labs Ltd, Bangalore, India, and protein powders were collected from Kiwi Nutritech, Chennai, India.

All subjects were treated according to the best available therapeutic strategy (standard management) or their condition. 

In addition, during the registry period, patients in the experimental group could use 25g power of JNS mixed with 250 ml of water a day, to alleviate their symptoms. The dosage was calculated based on previous reports done by the several researchers (16-17, 35). The aim of the supplement studies is to define the field of active evaluation of supplements and possible activation of reducing pain to improve the lifestyle by enhancing the symmetrical balances in the lower extremity or to other management plans. The following rules were adopted for the use of supplements in this study:

- As the supplements are not drugs, they are not prescribed but recommended for the improvement of the levels of outcome measures and lower anatomical parameters during the management of OA.
- The supplement is used in addition to the best management/ care, if available, based on appropriate international guidelines.
- There is no interference with any other treatment or preventive measure while using the supplement.
- A register is always maintained for the evaluation of these studies.
- The supplement is not very costly and easily available in the market without prescription.
- The patients considered as experimental subjects are those who have consumed the supplement continuously for week 12 as per register.
- A possible placebo effect is explained, and no placebo is used.
- Safety and tolerability were strictly evaluated.

**External study reviewers** *: *All results and data before and after the treatment were evaluated by an external reviewing panel consisting of a biostatistician, a radiologist, and a general medical practitioner, not in contract with the registry patients. 

**Evaluation of the interrater reliabilities and the degree of accuracy of data: **The agreement between the interrater reliabilities and the degree of accuracy for all the anatomical parameters of patients treated with JNS compare to control subjects according to the Cohen’s Kappa (*k*) values were evaluated. 

**Data collection and statistical analysis: **The primary aim was to investigate group diffences after the intensive treatment programme for 12 weeks with JNS and SYSADOA of the study participants.Data were summarized using descriptive statistics for continuous variables (e.g., mean, standard deviation, number of patients, minimum, maximum), frequency tables, and 95% confidence intervals.

 Statistical analyses were done using software (Graph Pad Prism, Version,5.0) with repeated measures for student-t test to determine significant values at p<0.05 level along with risk ratios, among two variables for measuring different improvement parameters of female-only and male-only patients separately. Intra- and inter-observer was determined using kappa statistics for anatomical parameters. An alpha level of 5% was established i.e., a p-value less than 0.05 was considered statistically significant. 

## Results

**Enrolment and baseline characteristics of patients: **Three-hundred thirty-four patients were screened out of 369 approaches. A total of 180 patients met the inclusion criteria and were enrolled. Finally, 108 patients were selected in experimental group and 72 were in control group (figure 1). The gender-wise analyses of age groups of 45-55, 55-65, and 65-75 years, for both the groups were shown in figures 2-3. In India, people with different ethnic groups have different types of food habits and take different kinds of food at the old age. They are also suffering with other disorders and take the help of different kinds of orthotics in OA. Therefore, in the baseline data analyses, Indian ethnic groups, food habits, other habits, multiple complaints are taken into consideration and shown in table 1. In both groups, women with OA changes are more prevailing than men by 14.38% and 15.28% with the age group of 65-75 years in the experimental and control groups respectively. All the patients were suffering from OA with severe pain, deterioration of daily lifestyle and restricted movement of lower extremity due to bone erosion and skeletal muscle damaged confirmed by radiological images. 

**Table 1:** Demographic data and baseline characteristics of the study subjects

**Characteristic**	**Experimental group**		**Control group**	
	**Female**	**Male**	**Female**	**Male**
No of subjects (%)	72 (66.67)	36 (33.33)	45 (62.50)	27 (37.50)
Mean age (SD) in years	64.89 (11.87)	64.37 (10.51)	63.88 (12.12)	65.12 (14.77)
Mean weight (SD) in kg	77.78 (6.17)	82.73 (6.19)	75.85 (7.32)	79.89 (7.77)
Mean height (SD) in meter	1.55 (0.92)	1.58 (0.88)	1.56 (0.93)	1.59 (0.98)
Mean BMI (SD) in kg/m²	32.51 (0.66)	33.51 (0.71)	30.77(0.82)	31.61 (0.98)
Mean symptom duration in years (SD)	6.82 (1.73)	7.72 (1.68)	6.82 (2.71)	7.17 (2.83)
**Indian ethnic group (%)**				
Bengali	24 (33.33)	8 (22.22)	8 (17.78)	6 (22.22)
Gujrati	9 (12.50)	5 (13.89)	5 (11.11)	3 (11.11)
Marwaree	8 (11.11)	4 (11.11)	6 (13.33)	4 (14.81)
Marathi	7 (9.72)	4 (11.11)	6 (13.33)	2 (7.41)
Tamil	6 (8.33)	5 (13.89)	5 (11.11)	3 (11.11)
Punjabi	6 (8.33)	4 (11.11)	6 (13.33)	5 (18.52)
Shindhi	7 (9.72)	3 (8.33)	4 (8.89)	2 (7.41)
North East India	5 (6.94)	3 (8.33)	5 (11.11)	2 (7.41)
**Dietary habits (%)**				
Vegetarian	37 (51.39)	21 (58.33)	31 (68.89)	15 (55.56)
Non- vegetarian	35 (48.61)	15(41.67)	14 (31.11)	12 (44.44)
**Other habits (%)**				
Smoking	12 (16.67)	6 (16.67)	11(24.44)	11 (40.74)
Drinking alcohol	8(11.11)	2 (5.56)	9 (13.33)	12(44.44)
Drinking tea and coffee	31 (43.06)	16 (44.44)	29 (54.44)	18 (66.67)
Chewing tobacco	7 (9.72)	1 (2.78)	5(11.11)	4 (14.81)
**Work status (%)**				
Employed fulltime	4 (5.56)	3 (8.33)	5 (11.11)	3 (11.11)
Employed part time	3 (4.17)	2 (5.56)	2 (4.44)	2 (7.41)
Housewife / Home- maker	58 (80.56)	0.00(0.00)	32 (71.11)	0.00(0.00)
Retired	4 (5.56)	23 (63.89)	4 (8.89)	19(70.37)
Self employed	3 (4.17)	8 (22.22)	2 (4.44)	3 (11.11)
**Marital status (%)**				
Single	2 (2.78)	2 (5.56)	2 (4.44)	1(3.70)
Married	49 (68.06)	21 (58.33)	35 (77.78)	21 (77.78)
Separated	2 ((2.78)	2 (5.56)	1 (2.22)	1(3.70)
Divorced	1 (1.39)	2 (5.56)	2 (4.44)	2(7.41)
Widowed	18 (25.00)	9 (25.00)	5 (11.11)	2(7.41)
**Multiple complaints or comorbiditie**s** (%)**				
Constipation	69 (95.83)	31 (86.11)	39 (886.67)	21(77.78)
Acidity and reflux	68 (94.44)	29 (80.56)	35(77.78)	19 (70.37)
Insomnia	67(93.06)	28 (77.78)	3168.89)	15 (55.56)
Varicose veins	31 (43.06)	21(58.33)	19 (42.22)	11(40.74)
Urinary incontinence	33 (45.83)	22 (61.11)	29 (64.44)	21(77.78)
Crepitus during knee flexion	66 (91.67)	31(86.11)	38 (84.44)	1866.67)
Morning stiffness (<30 min.)	65 (90.28)	28 (77.78)	36 (80.00)	17 (62.96)
**Measures taken to diminish pain and inflammation (%)**				
Kneecap uses	68 (94.44)	31 (86.11)	23 (51.11)	15 (55.56)
Lumbar belt uses	45 (62.50)	25 (69.44)	11 (24.44)	8 (29.63)
Paracetamol and NSAID use	71 (98.61)	24 (66.67)	41 (91.11)	23 (85.19)
Arthrocentesis (four months ago)	31 (43.06)	12 (33.33)	13 (28.89)	8 (29.63)
Use of hyaluronic acid injection	32 (44.44)	27 (75.00)	21 (46.67)	6 (22.22)
Use of corticosteroid injection	28 (38.89)	29 (80.56)	9 (20.00)	4 (14.81)
Massage with herbal or other gels	71 (98.61)	34 (94.44)	41 (91.11)	21 (77.78)
Homeopathic treatment	69 (95.83)	35(97.22)	39 (86.67)	23 (85.19)
Ayurvedic treatment	71 (98.61)	34 (94.44)	43 (95.56)	24 (88.89)
Stick/walker use	33 (45.83)	19 (52.78)	21 (46.67)	17 (62.96)

SD: standard deviation; KOA: knee-osteoarthritis; NSAID: non-steroidal anti-inflammatory drug.

Anatomical parameters: In the experimental group, the measurements of KGB, DAP and DBP were all reduced and DCM and DTM were all increased and observed to be symmetrical for both the legs at week 12 and were highly significant (p<0.0001) when compared to the control group (tables 2A-3B) But they were not significant and remained as asymmetrical in the control group at week 12 when compared to week 0 (tables 2A-3B).

**Table 2A:** Age group wise analyses of mean and standard divisions of KGB, DAP and DBP of 72 control patients, female (n=45), male (n=27) and 108 experimental patients, female (n=72), male (n=36) at week 0 and week 12

**Parameter**	**Age group**	**Gender**	**Control group**				**Experimental group**			
			**At week 0**		**At week12**		**At week 0**		**At week12**	
			**Right leg**	**Left leg**	**Right leg**	**Left leg**	**Right leg**	**Left leg**	**Right leg**	**Left leg**
			**Mean (SD)**	**Mean (SD)**	**Mean (SD)**	**Mean (SD)**	**Mean (SD)**	**Mean (SD)**	**Mean (SD)**	**Mean (SD)**
**KGB in cm**	**45-55**	Female (nᶜ=4, nᵉ=6)	6.61 (1.81)	6.94 (1.97)	6.60 (1.70)	6.90 (1.89)	6.60 (1.70)	6.70 (1.89)	3.05 (0.27)	3.05 (0.27)
		Male (nᶜ=3, nᵉ=4)	7.01 (1.98)	7.98 (2.39)	6.98 (1.86)	7.92 (2.16)	5.98 (1.86)	6.14 (2.16)	3.20 (0.27)	3.20 (0.27)
	**55-65**	Female (nᶜ=14, nᵉ=16)	9.20 (1.76)	10.12 (2.21)	8.68 (1.86)	9.73 (2.13)	7.68 (1.86)	7.83 (2.13)	3.56 (0.31)	3.56 (0.31)
		Male (nᶜ=8,nᵉ=10)	8.85 (2.21)	9.42 (2.31)	8.43 (2.36)	9.11 (2.59)	7.43 (2.36)	8.12 (2.59)	3.76 (0.18)	3.76 (0.18)
	**65-75**	Female (nᶜ=27,nᵉ=50)	11.08 (1.53)	11.78 (1.35)	10.76 (1.49)	11.56 (1.56)	9.95 (1.49)	10.40 (1.56)	3.78 (0.21)	3.78 (0.21)
		Male (nᶜ=16,nᵉ=22)	9.13 (1.19)	10.23 (1.52)	8.92 (1.24)	9.98 (1.41)	8.52 (1.24)	8.74 (1.41)	3.75 (0.21)	3.75 (0.21)
	**45-75**	**Female** **(n** **ᶜ** **=45,n** **ᵉ** **=72)**	**10.1 (1.63)**	**10.83 (1.72)**	**9.74 (1.63)**	**10.58 (1.79)**	**9.17 (1.63)**	**9.52 (1.79)**	**3.67 (0.25)**	**3.67 (0.25)**
		**Male** **(n** **ᶜ** **=27,n** **ᵉ** **=36)**	**8.81 (1.65)**	**9.74 (1.89)**	**8.56 (1.92)**	**9.49 (1.72)**	**7.94 (1.72)**	**8.28 (1.92)**	**3.69 (0.21)**	**3.69 (0.21)**
**DAP in cm**	**45-55**	Female (nᶜ=4, nᵉ=6)	42.70 (2.76)	42.31 (3.29)	42.59 (2.86)	42.18 (3.34)	42.59 (2.86)	42.18 (3.34)	40.45 (3.42)	40.45 (3.42)
		Male (nᶜ=3, nᵉ=4)	43.11 (3.87)	42.61 (3.19)	42.80 (4.04)	42.50 (3.32)	42.80 (4.04)	42.50 (3.32)	37.80 (1.64)	37.80 (1.64)
	**55-65**	Female (nᶜ=14, nᵉ=16)	42.68 (6.99)	42.88 (7.01)	42.57 (7.08)	42.80 (7.13)	42.57 (7.08)	42.80 (7.13)	40.13 (7.01)	40.13 (7.01)
		Male (nᶜ=8,nᵉ=10)	41.39 (4.11)	41.56 (3.71)	41.31 (4.20)	41.50 (3.90)	41.31 (4.20)	41.50 (3.90)	38.00 (3.96)	38.00 (3.96)
	**65-75**	Female (nᶜ=27,nᵉ=50)	42.90 (3.66)	43.32 (3.68)	42.81 (3.96)	43.26 (3.89)	41.91 (3.96)	41.99 (3.89)	39.64 (3.85)	39.64 (3.85)
		Male (nᶜ=16,nᵉ=22)	42.02 (4.98)	42.45 (4.58)	41.61 (4.68)	42.34 (4.71)	40.85 (4.68)	41.15 (4.71)	38.13 (3.77)	38.13 (3.77)
	**45-75**	**Female (n** **ᶜ** **=45,n** **ᵉ** **=72)**	**42.81 (4.89)**	**43.09 (4.94)**	**42.72 (5.07)**	**43.02 (5.09)**	**42.11 (5.07)**	**42.19 (5.09)**	**39.82 (5.02)**	**39.82 (5.02)**
		**Male** **(n** **ᶜ** **=27,n** **ᵉ** **=36)**	**41.95 (4.63)**	**42.2 (4.22)**	**41.65 (4.48)**	**42.11 (4.36)**	**41.19 (4.48)**	**41.4 (4.36)**	**38.06 (3.69)**	**38.06 (3.69)**
**DBP in cm**	**45-55**	Female (nᶜ=4, nᵉ=6)	33.79 (2.81)	34.34 (2.98)	33.73 (2.71)	34.18 (2.87)	33.73 (2.71)	33.68 (2.87)	32.65 (2.65)	32.65 (2.65)
		Male (nᶜ=3, nᵉ=4)	34.81 (2.55)	34.71 (2.21)	34.70 (2.66)	34.60 (2.38)	34.70 (2.66)	34.60 (2.38)	31.20 (0.84)	31.20 (0.84)
	**55-65**	Female (nᶜ=14, nᵉ=16)	35.49 (5.12)	36.41 (5.25)	35.41 (5.38)	36.32 (5.34)	35.30 (5.38)	35.52 (5.34)	34.30 (5.32)	34.30 (5.32)
		Male (nᶜ=8,nᵉ=10)	34.75 (2.15)	35.33 (2.41)	34.73 (2.55)	35.26 (2.52)	34.73 (2.55)	35.12 (2.52)	31.73 (2.98)	31.73 (2.98)
	**65-75**	Female (nᶜ=27,nᵉ=50)	35.09 (2.87)	35.92 (2.77)	35.01 (3.07)	35.85 (2.99)	34.66 (3.07)	34.59 (2.99)	33.51 (2.95)	33.51 (2.95)
		Male (nᶜ=16,nᵉ=22)	35.01 (3.86)	35.83 (4.37)	34.96 (4.04)	35.78 (4.18)	34.18 (4.04)	34.25 (4.18)	32.28 (3.92)	32.28 (3.92)
	**45-75**	**Female (n** **ᶜ** **=45,n** **ᵉ** **=72)**	**35.1 (3.71)**	**35.93 (3.74)**	**35.02 (3.91)**	**35.85 (3.87)**	**34.68 (3.91)**	**34.8 (3.87)**	**33.61 (3.83)**	**33.61 (3.83)**
		**Male** **(n** **ᶜ** **=27,n** **ᵉ** **=36)**	**34.91 (3.31)**	**35.56 (3.7)**	**34.86 (3.53)**	**35.49 (3.6)**	**34.39 (3.53)**	**34.53 (3.6)**	**32.01 (3.47)**	**32.01 (3.47)**

KGB: knee gap between the point of short head of the biceps femoris at the lateral knee and the surface of the bed; DAP: diameter of muscles connected with the knee joints 4 cm above the patella; DBP: diameter of muscles connected with the knee joints 4 cm below the patella; SD: standard deviation; nᶜ: number of patient in control group, nᵉ: number of patient in experimental group

**Table 2B:** Age group wise changes in knee gaps between biceps femoris-short head and surface of the bed (KGB) and diameters of muscles at 4 cm above and below the patella in control group, female (n=45) and male (n=27) from week 0-12, in experimental group female (n=72) and male (n=36) from week 0-12 and control-vs-experimental group at week 12

**Parameter**	**Age group**	**Gender**	**Changes in control group from week 0 to week 12**		**Changes in experimental group from week 0 to week 12**				**Changes in control -vs- experimental group at week 12**			
			**Right leg**	**Left leg**	**Right leg**		**Left leg**		**Right leg**		**Left leg**	
			**MD** **(95% CI)**	**MD** **(95% CI)**	**MD** **(95% CI)**	**p-value**	**MD** **(95% CI)**	**p-value**	**MD** **(95% CI)**	**p-value**	**MD** **(95% CI)**	**p-value**
**KGB in cm**	**45-55**	Female (nᶜ=4, nᵉ=6)	*-0.01 (-3.05, 3.03)	*-0.04 (-3.38, 3.30)	-3.55 (-4.63, -2.47)	<0.0001	-3.66 (-4.85, -2.45)	<0.0001	-3.55 (-5.13, -1.97)	0.0008	-3.85 (-5.61, -2.09)	0.001
		Male (nᶜ=3, nᵉ=4)	*-0.03 (-4.38, 4.32)	*-0.06 (-5.22, 5.10)	-2.78 (-4.72, -0.84)	0.011	-2.94 (-5.18, -0.69)	0.017	-3.78 (-6.13, -1.43)	0.009	-4.72 (-7.43, -2.01)	0.0066
	**55-65**	Female (nᶜ=14, nᵉ=16)	*-0.52 (-1.93, 0.89)	*-0.39 (-2.08, 1.30)	-4.12 (-4.81, -3.43)	<0.0001	-4.27 (-5.06, -3.48)	<0.0001	-5.12 (-6.00, -4.15)	<0.0001	-6.17 (-7.77, -5.07)	<0.0001
		Male (nᶜ=8,nᵉ=10)	*-0.42 (-2.87, 2.03)	*-0.31 (-2.94, 2.32)	-3.67 (-5.02, -2.31)	<0.0001	-4.36 (-5.85, -2.87)	<0.0001	-4.67 (-6.24, -3.09)	<0.0001	-5.35 (-7.08, -3.62)	<0.0001
	**65-75**	Female (nᶜ=27,nᵉ=50)	*-0.32 (-1.14, 0.51)	*-0.22 (-1.02, 0.58)	-6.19 (-6.58, -5.80)	<0.0001	-6.64 (-7.05, -6.23)	<0.0001	-6.98 (-7.41, -6.55)	<0.0001	-7.78 (-8.22, -7.34)	<0.0001
		Male (nᶜ=16,nᵉ=22)	*-0.21 (-1.09, 0.67)	*-0.25 (-1.31, 0.81)	-4.77 (-5.19, -4.35)	<0.0001	-4.99 (-5.46, -4.52)	<0.0001	-5.17 (-5.71, -4.13)	<0.0001	-6.23 (-6.85, -5.61)	<0.0001
	**45-75**	**Female** **(n** **ᶜ** **=45,n** **ᵉ** **=72)**	*-0.36 **(-1.04, 0.32)**	*-0.25 **(-0.98, 0.48)**	**-5.5** **(-5.88, -5.12)**	**<0.0001**	**-5.85** **(-6.27, -5.43)**	**<0.0001**	**-6.07** **(-6.46, -5.68)**	**<0.0001**	**-6.91** **(-7.33, -6.49)**	**<0.0001**
		**Male** **(n** **ᶜ** **=27,n** **ᵉ** **=36)**	*-0.25 **(-1.23, 0.73)**	*-0.25 **(-1.24, 0.74)**	**-4.25** **(-4.83, -3.67)**	**<0.0001**	**-4.59** **(-5.23, -3.95)**	**<0.0001**	**-4.87** **(-5.51, -4.23)**	**<0.0001**	**-5.80** **(-6.38, -5.22)**	**<0.0001**
**DAP in cm**	**45-55**	Female (nᶜ=4, nᵉ=6)	*-0.11 (-4.97, 4.75)	*-0.13 (-5.87, 5.61)	-2.14 (4.94, 0.66)	0.127	-1.73 (-4.74, 1.28)	0.244	-2.14 (-6.94, 2.66)	0.3335	-1.73 (-6.78, 3.32)	0.452
		Male (nᶜ=3, nᵉ=4)	*-0.31 (-9.28, 8.66)	*-0.11 (-7.49, 7.27)	-5.00 (-9.50, -0.50)	0.033	-4.70 (-8.52, -0.88)	0.022	-5.00 (-10.60, 0.60)	0.0703	-4.70 (-9.52, 0.12)	0.054
	**55-65**	Female (nᶜ=14, nᵉ=16)	*-0.11 (-5.58, 5.35)	*-0.08 (-5.57, 5.41)	-2.44 (-6.08, 1.20)	0.185	-2.67 (-6.32, 0.98)	0.149	-2.44 (-7.77, 2.84)	0.3519	-2.67 (-7.97, 2.63)	0.3107
		Male (nᶜ=8,nᵉ=10)	*-0.08 (-4.54, 4.38)	*0.06 (-4.14, 4.02)	-3.31 (-6.61, -0.01)	0.049	-3.50 (-6.68, -3.19)	0.032	-3.31 (-7.40, 0.78)	0.1055	-3.50 (-7.46, 0.46)	0.0791
	**65-75**	Female (nᶜ=27,nᵉ=50)	*-0.72 (-1.36, 2.80)	*-0.06 (-2.13, 2.01)	-2.27 (-3.71, -0.83)	0.002	-2.35 (-3.77, -0.93)	0.001	-3.17 (-5.02, -1.32)	0.001	-3.62 (-5.46, -1.78)	0.0002
		Male (nᶜ=16,nᵉ=22)	*-0.41 (-3.90, 3.08)	*-11 (-3.46, 3.24)	-2.72 (-4.72, -0.72)	0.008	-3.02 (-5.02, -1.01)	0.004	-3.48 (-6.26, -0.70)	0.0156	-4.21 (-7.01, -1.42)	0.0042
	**45-75**	**Female** **(n** **ᶜ** **=45,n** **ᵉ** **=72)**	*-0.09 **(-2.18, 1.99)**	*-0.07 **(-2.17, 2.03)**	**-2.29** **(-3.95, -0.83)**	**0.0073**	**-2.37** **(-4.04, -0.70)**	**0.0056**	**-2.90** **(-4.80, -1.00)**	**0.003**	**-3.20** **(-5.10, -1.30)**	**0.0011**
		**Male** **(n** **ᶜ** **=27,n** **ᵉ** **=36)**	*-0.30 **(-2.79, 2.19)**	*-0.09 **(-2.43, 2.25)**	**-3.13** **(-5.06, -1.20)**	**0.0019**	**-3.34** **(-5.24, -1.44)**	**0.0008**	**-3.59** **(-5.65, -1.53)**	**0.0009**	**-4.05** **(-6.08, -2.02)**	**0.0002**
**DBP in cm**	**45-55**	Female (nᶜ=4, nᵉ=6)	*-0.06 (-4.84, 4.71)	*-0.16 (-5.22, 4.90)	-1.08 (-3.46, 1.30)	0.356	-1.03 (-3.49, 1.42)	0.392	-1.08 (-5.06, 2.90)	0.5487	-1.53 (-5.61, 2.54)	0.4113
		Male (nᶜ=3, nᵉ=4)	*-0.11 (-6.02, 5.80)	*-0.11 (-5.32, 5.10)	-3.50 (-6.38, -0.62)	0.023	-3.40 (-6.01, -0.79)	0.017	-3.5 (-7.04, 0.04)	0.0519	-3.40 (-6.62, -0.18)	0.042
	**55-65**	Female (nᶜ=14, nᵉ=16)	*-0.08 (-4.16, 4.00)	*-0.09 (-4.20, 4.02)	-1.00 (-3.76, 1.76)	0.472	-1.22 (-3.97, 1.53)	0.379	-1.11 (-5.12, 2.90)	0.5751	-2.02 (-6.02, 1.98)	0.3092
		Male (nᶜ=8,nᵉ=10)	*-0.02 (-2.55, 2.51)	*-0.07 (-2.71, 2.57)	-3.00 (-5.24, -0.75)	0.011	-3.39 (-5.62, -1.16)	0.004	-3.00 (-5.82, -0.18)	0.0382	-3.53 (-6.33, -0.73)	0.0168
	**65-75**	Female (nᶜ=27,nᵉ=50)	*-0.08 (-1.70, 1.54)	*-0.07 (-1.64, 1.50)	-1.15 (-2.26, -0.04)	0.042	-1.08 (-2.17, 0.01)	0.052	-1.5 (-2.92, -0.08)	0.0392	-2.34 (-3.75, -0.93)	0.0015
		Male (nᶜ=16,nᵉ=22)	*-0.05 (-2.90, 2.80)	*-0.05 (-3.14, 3.04)	-1.90 (-3.77, -0.03)	0.046	-1.97 (-3.87, -0.06)	0.043	-2.68 (-5.33, -0.03)	0.0473	-3.50 (-6.19, -0.81)	0.0121
	**45-75**	**Female** **(n** **ᶜ** **=45,n** **ᵉ** **=72)**	*-0.08 **(-1.68, 1.52)**	*-0.14 **(-1.73, 1.45)**	**-1.07** **(-2.34, 0.21)**	**0.0994**	**-1.19** **(-2.46, 0.08)**	**0.0657**	**-1.41** **(-2.86, 0.04)**	**0.0571**	**-2.24** **(-3.69, -0.79)**	**0.0027**
		**Male** **(n** **ᶜ** **=27,n** **ᵉ** **=36)**	*-0.05 **(-1.92, 1.82)**	*-0.07 **(-2.06, 1.92)**	**-2.38** **(-4.03, -0.73)**	**0.0052**	**-2.52** **(-4.18, -0.86)**	**0.0035**	**-2.85** **(-4.63, -1.07)**	**0.0022**	**-2.48** **(-4.28, -0.68)**	**0.0076**

KGB: knee gap between the point of short head of the biceps femoris at the lateral knee and the surface of the bed; DAP: diameter of muscles connected with the knee joints 4 cm above the patella; DBP: diameter of muscles connected with the knee joints 4 cm below the patella; MD: mean difference; CI: confidence interval; *p = not significant; nᶜ: number of patient in control group, nᵉ: number of patient in experimental group

**Table 3A:** Age group wise analyses of mean and standard divisions of DTM and DCM of 72 control patients, female (n=45), male (n=27) and 108 experimental patients, female (n=72), male (n=36) at week 0 and week 12

**Parameter**	**Age group**	**Gender**	**Control group**				**Experimental group**			
			**At week 0**		**At week12**		**At week 0**		**At week12**	
			**Right leg**	**Left leg**	**Right leg**	**Left leg**	**Right leg**	**Left leg**	**Right leg**	**Left leg**
			**Mean (SD)**	**Mean (SD)**	**Mean (SD)**	**Mean (SD)**	**Mean (SD)**	**Mean (SD)**	**Mean (SD)**	**Mean (SD)**
**DTM in cm**	**45-55**	Female (nᶜ=4, nᵉ=6)	46.98 (3.34)	47.68 (3.68)	47.00 (3.28)	47.79 (3.58)	47.00 (3.28)	46.77 (3.58)	48.14 (3.46)	48.14 (3.46)
		Male (nᶜ=3, nᵉ=4)	45.47 (3.15)	46.21 (2.74)	45.50 (3.04)	46.25 (2.62)	45.50 (3.04)	46.00 (2.62)	49.80 (0.67)	49.80 (0.67)
	**55-65**	Female (nᶜ=14, nᵉ=16)	49.29 (7.35)	49.38 (7.55)	49.32 (7.51)	49.40 (7.57	48.48 (7.51)	48.50 (7.57)	49.45 (7.55)	49.45 (7.55)
		Male (nᶜ=8,nᵉ=10)	47.09 (4.23)	47.23 (3.91)	47.11 (4.03)	47.25 (3.84)	45.69 (4.03)	45.73 (3.84)	48.96 (3.25)	48.96 (3.25)
	**65-75**	Female (nᶜ=27,nᵉ=50)	49.31 (4.44)	49.45 (4.12)	49.38 (4.21)	49.50 (4.05)	48.37 (4.21)	48.46 (4.09)	49.35 (4.66)	49.35 (4.66)
		Male (nᶜ=16,nᵉ=22)	46.69 (4.72)	47.36 (4.81)	46.71 (4.70)	47.40 (4.70)	46.76 (4.70)	47.04 (4.70)	49.71 (4.08)	49.71 (4.08)
	**45-75**	**Female** **(n** **ᶜ** **=45,n** **ᵉ** **=72)**	**49.1 (5.44)**	**49.27 (5.4)**	**49.15 (5.4)**	**49.32 (5.37)**	**48.28 (5.4)**	**48.32 (5.39)**	**49.27 (5.64)**	**49.27 (5.64)**
		**Male** **(n** **ᶜ** **=27,n** **ᵉ** **=36)**	**46.71 (4.45)**	**47.22 (4.39)**	**46.73 (4.37)**	**47.25 (4.3)**	**46.32 (4.37)**	**46.54 (4.3)**	**49.48 (3.65)**	**49.48 (3.65)**
**DCM in cm**	**45-55**	Female (nᶜ=4, nᵉ=6)	33.72 (2.61)	33.97 (2.87)	33.75 (2.52)	34.01 (2.98)	33.00 (2.52)	33.23 (3.00)	32.14 (2.68)	32.14 (2.68)
		Male (nᶜ=3, nᵉ=4)	33.98 (0.72)	34.37 (0.99)	34.00 (0.61)	34.40 (1.02)	34.00 (0.61)	34.40 (1.02)	35.10 (0.22)	35.10 (0.22)
	**55-65**	Female (nᶜ=14, nᵉ=16)	34.10 (5.32)	34.88 (5.21)	34.12 (5.21)	34.89 (5.36)	33.99 (5.21)	34.00 (5.36)	33.97 (5.26)	33.97 (5.26)
		Male (nᶜ=8,nᵉ=10)	34.42 (2.29)	34.70 (2.31)	34.43 (2.28)	34.72 (2.33)	34.38 (2.28)	34.58 (2.33)	35.54 (2.16)	35.54 (2.16)
	**65-75**	Female (nᶜ=27,nᵉ=50)	34.04 (3.14)	34.96 (2.98)	34.03 (3.03)	34.95 (3.02)	33.69 (3.03)	33.64 (3.02)	34.47 (3.13)	34.47 (3.13)
		Male (nᶜ=16,nᵉ=22)	34.86 (3.74)	35.04 (3.68)	34.84 (3.63)	35.01 (3.58)	34.31 (3.63)	34.47 (3.58)	35.18 (3.77)	35.18 (3.77)
	**45-75**	**Female** **(n** **ᶜ** **=45,n** **ᵉ** **=72)**	**34.03 (3.91)**	**34.85 (3.81)**	**34.03 (3.81)**	**34.85 (3.9)**	**33.7 (3.81)**	**33.72 (3.9)**	**34.11 (3.89)**	**34.11 (3.89)**
		**Male** **(n** **ᶜ** **=27,n** **ᵉ** **=36)**	**34.63 (3.17)**	**34.86 (3.14)**	**34.63 (3.09)**	**34.86 (3.08)**	**34.3 (3.09)**	**34.5 (3.08)**	**35.28 (3.16)**	**35.28 (3.16)**

DTM: diameter of muscles at the thighs; DCM: diameter of muscles at the calves; SD: standard deviation; nᶜ: number of patient in control group, nᵉ: number of patient in experimental group

**Table 3B:** Age group wise changes in diameters of muscles at thigh and calf in control group, female (n=45) and male (n=27) from week o -12, in experimental group female (n=72) and male (n=36) from week 0-12 and control-vs-experimental group at week 12

**Parameter**	**Age group**	**Gender**	**Changes in control group from week 0 to week 12**		**Changes in experimental group from week 0 to week 12**				**Changes in control -vs- experimental group at week 12**			
			**Right leg**	**Left leg**	**Right leg**		**Left leg**		**Right leg**		**Left leg**	
			**MD** **(95% CI)**	**MD** **(95% CI)**	**MD** **(95% CI)**	**p-value**	**MD** **(95% CI)**	**p-value**	**MD** **(95% CI)**	**p-value**	**MD** **(95% CI)**	**p-value**
**DTM in cm**	**45-55**	Female (nᶜ=4, nᵉ=6)	*0.02 (-5.71, 5.74)	*0.11 (-6.17, 6.39)	1.14 (-1.86, 4.14)	0.437	1.37 (-1.76, 4.51)	0.372	1.14 (-3.91, 6.19)	0.616	0.35 (-4.87, 5.57)	0.880
		Male (nᶜ=3, nᵉ=4)	*0.03 (-6.99, 7.05)	*0.04 (-6.04, 6.12)	4.3 (1.39, 7.51)	0.014	3.8 (1.01, 6.59)	0.014	4.3 (0.39, 8.21)	0.036	3.55 ( 0.14, 6.96)	0.044
	**55-65**	Female (nᶜ=14, nᵉ=16)	*0.03 (-5.74, 5.80)	*0.02 (-5.85, 5.89)	1.48 (-2.41, 5.37)	0.449	1.46 (-2.45, 5.37)	0.457	0.13 (-5.52, 5.78)	0.962	0.05 (-5.62, 5.72)	0.985
		Male (nᶜ=8,nᵉ=10)	*0.02 (-4.40, 4.45)	*0.02 (-4.13, 4.18)	3.27 (0.31, 6.23)	0.032	3.23 (0.35, 6.11)	0.029	1.85 (-1.78, 5.48)	0.296	1.71 (-1.83, 5.25)	0.321
	**65-75**	Female (nᶜ=27,nᵉ=50)	*0.07 (-2.29, 2.43)	*0.05 (-2.18, 2.28)	0.98 (-0.65, 2.61)	0.237	0.89 (-0.72, 2.5)	0.277	0.5 (-1.65, 2.64)	0.643	0.38 (-1.74, 2.50)	0.722
		Male (nᶜ=16,nᵉ=22)	*0.02 (-3.38, 3.42)	*0.04 (-3.39, 3.47)	2.95 (0.88, 5.02)	0.005	2.67 (0.6, 4.74)	0.012	3 (0.11, 5.89)	0.042	2.31 (-0.59, 5.21)	0.114
	**45-75**	**Female** **(n** **ᶜ** **=45,n** **ᵉ** **=72)**	*-0.05 **(-2.22, 2.32)**	*0.05 **(-2.21, 2.31)**	**0.99** **(-0.83, 2.81)**	**0.283**	**0.95** **(-0.87, 2.77)**	**0.303**	**0.12** **(-1.97, 2.21)**	**0.909**	**-0.05** **(-2.13, 2.03)**	**0.962**
		**Male** **(n** **ᶜ** **=27,n** **ᵉ** **=36)**	*0.02 **(-2.39, 2.43)**	*-0.97 **(-3.34, 1.40)**	**3.16** **(1.27, 5.05)**	**0.001**	**2.94** **(1.06, 4.81)**	**0.002**	**2.75** **(0.72, 4.77)**	**0.008**	**2.23** **(0.22, 4.24)**	**0.029**
**DCM in cm**	**45-55**	Female (nᶜ=4, nᵉ=6)	*0.03 (-4.39, 4.49)	*0.04 (-5.02, 5.10)	-0.86 (-3.17, 1.45)	0.447	-1.09 (-3.62, 1.44)	0.379	0.38 (-3.51, 4.29)	0.823	0.13 (-4.03, 4.29)	0.944
		Male (nᶜ=3, nᵉ=4)	*0.02 (-1.49, 1.53)	*0.03 (-2.25, 2.31)	1.01 (0.34, 1.67)	0.008	0.61 (-0.47, 1.69)	0.227	1.1 (0.27, 1.93)	0.018	0.70 (-0.61, 2.01)	0.228
	**55-65**	Female (nᶜ=14, nᵉ=16)	*0.02 (-4.07, 4.11)	*0.01 (-4.10, 4.12)	-0.02 (-2.72, 2.68)	0.988	-0.03 (-2.78, 2.71)	0.983	0.85 (-3.07, 4.77)	0.660	0.08 (-3.90, 4.06)	0.967
		Male (nᶜ=8,nᵉ=10)	*0.01 (-2.44, 2.46)	*0.02 (-2.47, 2.51)	1.16 (-0.64, 2.96)	0.195	0.96 (-0.86, 2.78)	0.287	1.16 (-1.06, 3.38)	0.285	0.82 (-1.48, 3.12)	0.461
	**65-75**	Female (nᶜ=27,nᵉ=50)	*-0.01 (-1.69, 1.67)	*-0.01 (-1.65, 1.63)	0.78 (-0.35, 1.91)	0.175	0.83 (-0.3, 1.96)	0.149	0.44 (-1.03, 1.91)	0.553	0.13 (-1.95, 1.59)	0.871
		Male (nᶜ=16,nᵉ=22)	*-0.02 (-2.68, 2.64)	*-0.03 (-2.65, 2.59)	0.87 (-0.87, 2.61)	0.322	0.71 (-1.02, 2.44)	0.415	0.34 (-2.13, 2.81)	0.782	0.17 (-2.29, 2.63)	0.889
	**45-75**	**Female** **(n** **ᶜ** **=45,n** **ᵉ** **=72)**	*0.00 **(-1.62, 1.62)**	*0.00 **(-1.61, 1.61)**	**0.41** **(-0.86, 1.68)**	**0.523**	**0.39** **(-0.89, 1.67)**	**0.549**	**0.08** **(-1.37, 1.53)**	**0.913**	**-0.74** **(-2.21, 0.72)**	**0.319**
		**Male** **(n** **ᶜ** **=27,n** **ᵉ** **=36)**	*0.00 **(-1.71, 1.71)**	*0.00 **(-1.70, 1.70)**	**0.98** **(-0.49, 2.45)**	**0.187**	**0.78** **(-0.69, 2.25)**	**0.292**	**0.65** **(-0.94, 2.24)**	**0.417**	**0.42** **(-1.17,2.01)**	**0.599**

DTM: diameter of muscles at the thighs; DCM: diameter of muscles at the calves; MD: mean difference; CI: confidence interval; *p = not significant; nᶜ: number of patient in control group, nᵉ: number of patient in experimental group

In the experimental group, the mean ±SD values of SLR and KFS at week 12 all increased, decreased for KES and observed to be all symmetrical for both the legs. All the differences were highly significant (p<0.0001) when compared to the female-only and man-only of control group (table 4A-B) But the mean ±SD values were not significant and remained as asymmetrical for female-only and male-only of control group at week-12 compared to week 0 (table 4A-B)

**Table 4A:** Age group wise analyses of mean and standard divisions of SLR, KFS, and KES of 72 control patients, female (n=45), male (n=27) and 108 experimental patients, female (n=72), male (n=36) at week 0 and week 12

**Parameter**	**Age group**	**Gender**	**Control group**				**Experimental group**			
			**At week 0**		**At week12**		**At week 0**		**At week12**	
			**Right leg**	**Left leg**	**Right leg**	**Left leg**	**Right leg**	**Left leg**	**Right leg**	**Left leg**
			**Mean (SD)**	**Mean (SD)**	**Mean (SD)**	**Mean (SD)**	**Mean (SD)**	**Mean (SD)**	**Mean (SD)**	**Mean (SD)**
**SLR in degree**	**45-55**	Female (nᶜ=4, nᵉ=6)	43.58 (14.48)	42.18 (14.04)	43.55 (14.59)	42.12 (14.19)	43.55 (14.59)	43.45 (14.19)	75.45 (2.16)	75.45 (2.16)
		Male (nᶜ=3, nᵉ=4)	41.27 (17.51)	38.20 (17.49)	41.23 (17.71)	38.17 (17.62)	53.20 (17.71)	52.40 (17.62)	75.00 (1.58)	75.00 (1.58)
	**55-65**	Female (nᶜ=14, nᵉ=16)	42.80 (13.98)	39.55 (14.99)	42.67 (14.05)	39.39 (15.44)	42.67 (14.05)	41.60 (15.44)	74.03 (2.71)	74.03 (2.71)
		Male (nᶜ=8,nᵉ=10)	46.15 (12.71)	44.36 (12.83)	46.12 (12.93)	44.25 (12.91)	49.65 (12.93)	51.12 (12.91)	72.54 (1.98)	72.54 (1.98)
	**65-75**	Female (nᶜ=27,nᵉ=50)	34.88 (4.42)	32.18 (4.98)	34.77 (4.45)	32.09 (5.09)	34.53 (4.45)	33.96 (5.09)	71.33 (1.78)	71.33 (1.78)
		Male (nᶜ=16,nᵉ=22)	38.77 (8.81)	36.90 (8.53)	38.65 (8.70)	36.81 (8.56)	40.94 (8.70)	39.44 (8.56)	71.17 (1.36)	71.17 (1.36)
	**45-75**	**Female** **(n** **ᶜ** **=45,n** **ᵉ** **=72)**	**38.12 (9.55)**	**35.36 (10.11)**	**38.01 (9.6)**	**35.25 (10.37)**	**37.86 (9.6)**	**37.18 (10.37)**	**72.54 (2.14)**	**72.54 (2.14)**
		**Male** **(n** **ᶜ** **=27,n** **ᵉ** **=36)**	**41.29 (11.14)**	**39.34 (11.05)**	**41.2 (11.19)**	**39.25 (11.11)**	**44.81 (11.19)**	**44.23 (11.11)**	**71.94 (1.6)**	**71.94 (1.6)**
**KFS in degree**	**45-55**	Female (nᶜ=4, nᵉ=6)	107.80 (2.73)	104.67 (4.19)	107.65 (3.63)	104.54 (4.10)	110.09 (13.63)	110.55 (14.10)	138.91 (1.22)	138.91 (1.22)
		Male (nᶜ=3, nᵉ=4)	110.58 (4.91)	102.40 (1.81)	110.49 (4.82)	102.25 (1.79)	103.20 (4.82)	102.20 (1.79)	140.00 (0.03)	140.00 (0.03)
	**55-65**	Female (nᶜ=14, nᵉ=16)	108.20 (3.64)	105.85 (3.91)	108.12 (3.53)	105.76 (3.88)	109.77 (13.53)	110.37 (13.88)	136.97 (1.10)	136.97 (1.10)
		Male (nᶜ=8,nᵉ=10)	109.30 (6.21)	114.37 (5.81)	109.17 (6.78)	114.25 (5.72)	114.15 (16.78)	115.85 (15.72)	139.00 (4.26)	139.00 (4.26)
	**65-75**	Female (nᶜ=27,nᵉ=50)	104.98 (4.61)	103.25 (4.11)	104.81 (4.73)	103.11 (4.15)	105.03 (4.73)	104.93 (4.15)	136.36 (0.97)	136.36 (0.97)
		Male (nᶜ=16,nᵉ=22)	110.15 (10.41)	104.78 (10.59)	110.01 (10.62)	104.65 (10.61)	110.33 (10.62)	110.64 (10.61)	137.58 (4.40)	137.58 (4.40)
	**45-75**	**Female** **(n** **ᶜ** **=45,n** **ᵉ** **=72)**	**106.23 (4.19)**	**104.19 (4.06)**	**106.09 (4.3)**	**104.06 (4.06)**	**106.95 (9.32)**	**107.12 (9.38)**	**136.78 (1.04)**	**136.78 (1.04)**
		**Male** **(n** **ᶜ** **=27,n** **ᵉ** **=36)**	**109.61 (8.9)**	**107.55 (8.84)**	**109.79 (9.16)**	**107.42 (8.84)**	**110.88 (12.54)**	**111.51 (12.03)**	**138.24 (4.15)**	**138.24 (4.15)**
**KES in degree**	**45-55**	Female (nᶜ=4, nᵉ=6)	20.18 (4.41)	17.96 (4.52)	20.25 (4.38)	18.03 (4.43)	16.94 (4.38)	17.07 (4.43)	9.50 (0.45)	9.50 (0.45)
		Male (nᶜ=3, nᵉ=4)	18.55 (4.57)	17.75 (3.78)	18.65 (4.58)	18.01 (3.99)	13.70 (4.58)	13.60 (4.99)	8.50 (0.21)	8.50 (0.21)
	**55-65**	Female (nᶜ=14, nᵉ=16)	19.54 (2.91)	17.38 (3.71)	19.65 (2.86)	17.45 (3.66)	17.49 (2.86)	17.85 (3.66)	8.88 (0.25)	8.88 (0.25)
		Male (nᶜ=8,nᵉ=10)	18.01 (5.81)	17.33 (5.01)	18.11 (5.75)	17.42 (5.02)	15.19 (5.75)	14.19 (5.02)	8.88 (0.55)	8.88 (0.55)
	**65-75**	Female (nᶜ=27,nᵉ=50)	17.88 (2.61)	18.35 (2.71)	18.05 (2.58)	18.47 (2.64)	18.05 (2.58)	17.89 (2.64)	8.35 (0.43)	8.35 (0.43)
		Male (nᶜ=16,nᵉ=22)	17.68 (2.29)	16.92 (2.39)	17.79 (2.31)	17.03 (2.42)	17.36 (2.31)	17.47 (2.42)	9.11 (0.73)	9.11 (0.73)
	**45-75**	**Female** **(n** **ᶜ** **=45,n** **ᵉ** **=72)**	**18.6 ** **(2.91)**	**18.01 ** **(3.24)**	**18.74 (2.87)**	**18.11 (3.18)**	**17.78 (2.87)**	**17.8 (3.18)**	**8.62 (0.39)**	**8.62 (0.39)**
		**Male** **(n** **ᶜ** **=27,n** **ᵉ** **=36)**	**17.86 (3.94)**	**17.12 ** **(3.54)**	**17.97 (3.92)**	**17.24 (3.57)**	**16.36 (3.92)**	**16.11 (3.68)**	**8.96 (0.65)**	**8.96 (0.65)**

SLR: straight legs raising in supine; KFS: angle of knee flexion in supine; KES: angle of knee extension in supine; SD: standard deviation; nᶜ: number of patient in control group, nᵉ: number of patient in experimental group

**Table 4B:** Age group wise changes in angles of straight leg raising, knee flexion and knee extension in supine in control group, female (n=45) and male (n=27) from week 0 -12, in experimental group female (n=72) and male (n=36) from week 0-12 and control-vs-experimental group at week 12

**Parameter**	**Age group**	**Gender**	**Changes in control group from week 0 to week 12**		**Changes in experimental group from week 0 to week 12**				**Changes in control -vs- experimental group at week 12**			
			**Right leg**	**Left leg**	**Right leg**		**Left leg**		**Right leg**		**Left leg**	
			**MD ** **(95% CI)**	**MD** ** (95% CI)**	**MD ** **(95% CI)**	**P-value**	**MD ** **(95% CI)**	**P-value**	**MD ** **(95% CI)**	**p-value**	**MD ** **(95% CI)**	**P-value**
**SLR in degree**	**45-55**	Female (nᶜ=4, nᵉ=6)	*-0.03 (-25.18, 25.12)	*0.06 (-24.48, 24.36)	31.90 (22.62, 41.18)	<0.0001	32 (22.97, 41.03)	<0.0001	31.9 (18.36, 45.44)	0.0006	33.33 (20.15, 46.51)	0.0004
		Male (nᶜ=3, nᵉ=4)	*-0.04 (-39.96, 39.88)	*-0.03 (-39.83, 39.77)	21.80 (3.46, 40.14)	0.025	22.6 (4.36, 40.84)	0.025	33.77 (11.65, 55.89)	0.0111	36.83 (14.82, 58.84)	0.0077
	**55-65**	Female (nᶜ=14, nᵉ=16)	*-0.13 (-11.02, 10.76)	*-0.16 (-11.98, 11.66)	31.36 (26.13, 36.59)	<0.0001	32.43 (26.70, 38.16)	<0.0001	31.36 (24.04, 38.69)	<0.0001	34.64 (26.61, 42.67)	<0.0001
		Male (nᶜ=8,nᵉ=10)	*-03 (-13.78, 13.72)	*-0.11 (-13.91, 13.69)	22.89 (15.40, 30.38)	<0.0001	21.42 (13.94, 28.90)	<0.0001	26.42 (17.69, 35.15)	<0.0001	28.29 (19.57, 37.01)	<0.0001
	**65-75**	Female (nᶜ=27,nᵉ=50)	*-0.11 (-2.53, 2.31)	*-0.09 (-2.84, 2.66)	36.80 (35.53, 38.05)	<0.0001	37.37 (35.97, 38.77)	<0.0001	36.56 (35.14, 37.98)	<0.0001	39.24 (37.66, 40.82)	<0.0001
		Male (nᶜ=16,nᵉ=22)	*-0.12 (-6.44, 6.21)	*-0.09 (-6.26, 6.08)	30.23 (27.30, 33.16)	<0.0001	31.73 (28.85, 34.61)	<0.0001	32.52 (28.71, 36.33)	<0.0001	34.36 (30.61, 38.11)	<0.0001
	**45-75**	**Female (n** **ᶜ** **=45,n** **ᵉ** **=72)**	*-0.11 **(-4.12, 3.90)**	*-0.11 **(-4.40, 4.18)**	**34.68** **(32.39, 36.97)**	**<0.0001**	**35.36** **(32.89, 37.83)**	**<0.0001**	**34.53** **(32.21, 36.85)**	**<0.0001**	**37.29 (34.79,39.79)**	**<0.0001**
		**Male (n** **ᶜ** **=27,n** **ᵉ** **=36)**	*-0.09 **(-6.19, 6.01)**	*-0.09 **(-6.14, 5.96)**	**27.13** **(23.37, 30.89)**	**<0.0001**	**27.71** **(23.98, 31.44)**	**<0.0001**	**30.74** **(26.97, 34.51)**	**<0.0001**	**32.69** **(28.95, 36.43)**	**<0.0001**
**KFS in degree**	**45-55**	Female (nᶜ=4, nᵉ=6)	*-0.15 (-5.71, 5.41)	*-0.13 (-7.31, 7.05)	28.82 (20.21, 37.43)	<0.0001	28.36 (19.46, 37.26)	<0.0001	31.26 (18.75, 43.77)	0.0004	33.37 (20.44, 46.30)	0.0003
		Male (nᶜ=3, nᵉ=4)	*-0.09 (-11.12, 10.94)	*-0.15 (-4.23, 3.93)	36.80 (31.83, 41.77)	<0.0001	37.80 (35.95, 39.65)	<0.0001	29.51 (23.52, 35.49)	0.0001	37.75 (35.53,39.97)	<0.0001
	**55-65**	Female (nᶜ=14, nᵉ=16)	*-0.08 (-2.86, 2.71)	*-0.09 (-3.12, 2.94)	27.20 (22.24, 32.16)	<0.0001	26.60 (21.51, 31.69)	<0.0001	28.85 (21.91, 35.79)	<0.0001	31.21 (24.09, 38.33)	<0.0001
		Male (nᶜ=8,nᵉ=10)	*-0.13 (-7.10, 6.84)	*-0.12 (-6.30, 6.06)	24.85 (14.94, 34.76)	<0.0001	23.15 (13.82, 32.47)	<0.0001	26.86 (15.22, 38.44)	0.0002	24.75 (13.81,35.69)	0.0002
	**65-75**	Female (nᶜ=27,nᵉ=50)	*-0.17 (-2.72, 2.38)	*-0.14 (-2.40, 2.12)	31.33 (30.07, 32.59)	<0.0001	31.43 (30.32, 32.53)	<0.0001	31.55 (30.17, 32.93)	<0.0001	33.25 (32.03, 34.47)	<0.0001
		Male (nᶜ=16,nᵉ=22)	*-0.14 (-7.32, 7.45)	*-0.13 (-7.78, 7.52)	27.25 (23.43, 31.07)	<0.0001	26.94 (23.12, 30.76)	<0.0001	27.57 (22.48, 32.66)	<0.0001	32.93 (27.85,38.01)	<0.0001
	**45-75**	**Female (n** **ᶜ** **=45,n** **ᵉ** **=72)**	*-0.14 **(-1.92,1.64)**	*-0.10 **(-1.80, 1.60)**	**29.83** **(27.64, 32.01)**	**<0.0001**	**29.66** **(27.46, 31.86)**	**<0.0001**	**30.69** **(29.64, 31.74)**	**<0.0001**	**32.72** **(31.72, 33.71)**	**<0.0001**
		**Male (n** **ᶜ** **=27,n** **ᵉ** **=36)**	*0.18 **(-4.75, 5.11)**	*-0.13 **(-4.96, 4.70)**	**27.36** **(22.97, 31.75)**	**<0.0001**	**26.73** **(22.50, 30.96)**	**<0.0001**	**28.45** **(25.01, 31.89)**	**<0.0001**	**30.82** **(27.47, 34.16)**	**<0.0001**
**KES in degree**	**45-55**	Female (nᶜ=4, nᵉ=6)	*0.07 (-7.53, 7.67)	*0.28 (-7.46, 8.02)	-7.34 (-10.11, -4.57)	<0.0001	-7.47 (-10.27,-4.67)	<0.0001	-10.75 (-14.78, -6.72)	0.0003	-8.53 (-12.60, -4.46)	0.0013
		Male (nᶜ=3, nᵉ=4)	*0.10 (-8.00, 8.20)	*0.26 (-8.55, 9.07)	-5.2 (-9.93, -0.47)	0.035	-5.10 (-10.25, 0.05)	0.035	-10.15 (-15.86, -4.45)	0.0059	-9.51 (-15.71,-3.31)	0.011
	**55-65**	Female (nᶜ=14, nᵉ=16)	*0.11 (-2.85, 3.07)	*0.07 (-2.79, 2.93)	-8.61 (-9.66, -7.56)	<0.0001	-8.97 (-10.31, -7.63)	<0.0001	-10.77 (-12.24, -9.3)	<0.0001	-8.57 (-10.46, -6.67)	<0.0001
		Male (nᶜ=8,nᵉ=10)	*0.10 (-6.10, 6.30)	*0.09 (-5.29, 5.47)	-6.31 (-9.62, -3.00)	0.0006	-5.31 (-8.20, -2.42)	0.0006	-9.23 (-13.08, -5.39)	0.0001	-8.54 (-11.90, -5.17)	0.0001
	**65-75**	Female (nᶜ=27,nᵉ=50)	*0.17 (-1.24, 1.58)	*0.12 (-1.34, 1.58)	-9.7 (-10.38, -9.02)	<0.0001	-9.54 (-10.24, -8.84)	<0.0001	-9.7 (-10.44, -8.96)	<0.0001	-10.12 (-10.88, -9.36)	<0.0001
		Male (nᶜ=16,nᵉ=22)	*0.11 (-1.55, 1.77)	*0.11 (-1.63, 1.85)	-8.25 (-9.05, -7.44)	<0.0001	-8.31 (-9.15, -7.47)	<0.0001	-8.68 (-9.74, -7.62)	<0.0001	-7.92 (-9.03, -6.82)	<0.0001
	**45-75**	**Female (n** **ᶜ** **=45,n** **ᵉ** **=72)**	*0.14 **(-1.07, 1.35)**	*0.10 **(-1.24, 1.44)**	**-9.16** **(-9.83, -8.48)**	**<0.0001**	**-9.18** **(-9.93, -8.43)**	**<0.0001**	**-10.12** **(-10.79, -9.44)**	**<0.0001**	**9.48** **(-10.24,-8.74)**	**<0.0001**
		**Male (n** **ᶜ** **=27,n** **ᵉ** **=36)**	*0.11 **(-2.04, 2.26)**	*0.12 **(-1.82, 2.06)**	**-7.40** **(-8.72, -6.08)**	**<0.0001**	**-7.15** **(-8.39, -5.91)**	**<0.0001**	**-9.01** **(-10.34, -7.68)**	**<0.0001**	**-8.28** **(-9.49, -7.07)**	**<0.0001**

SLR: straight legs raising in supine; KFS: angle of knee flexion in supine; KES: angle of knee extension in supine; SD: standard deviation; *p = not significant; nᶜ: number of patient in control group, nᵉ: number of patient in experimental group

Table 5 shows that the risk ratios for all the studied anatomical parameters for both the legs of experimental subjects (female and male) treated with JNS were highly significant (p<0.0001) compared to the control subjects (Figure 5). 

Table 6 shows that the correlation coefficients of aberrant anatomical parameters between week 0 and week 12 were all positively correlated for the subjects of experimental group.

**Improvements on bone health as per radiological images as assessed by Kellgren-Lawrance grading scale: **All the anterior-posterior (AP) views of the x-ray reports of 180 patients with OA at the baseline exhibited degenerative changes, particularly in the medial tibiofemoral compartment, with marked joint space narrowing with osteophytes and bilateral varus/valgus deformities. The AP view of X-rays for bilateral knee joints of 108 patients after twelve weeks of treatment with JNS indicated substantial improvements on degenerative changes as well as bone health as assessed under K-L grading scale upgraded between ≥ 2 and ≥3 shown in table 7 and the balance of 72 patients treated with SYSADOA having further deterioration on bone health. X-rays images of two such patients suffering from pain in the knee joint before and after the treatment with supplements were depicted in figure 8.

**Table 5:** Analysis of risk ratios of lower extremities of 108 patients, female (*n*=72), and male (*n*=36), using Jumpstart Nutrition® supplement compared to 72 control subjects, female (*n*=45), and male (*n*=27) at week 12

		**RIGHT LEG**				**LEFT LEG**			
		**RR**	**95% CI**		**P-value**	**RR**	**95% CI**		**P-value**
			**Lower**	**Upper**			**Lower**	**Upper**	
**KGB**	**Female**	0.204	0.14	0.31	<0.0001	0.211	0.14	0.32	<0.0001
	**Male**	0.12	0.06	0.25	<0.0001	0.122	0.06	0.26	<0.0001
**DAP**	**Female**	0.594	0.45	0.77	0.0001	0.611	0.47	0.81	0.0004
	**Male**	0.143	0.06	0.34	<0.0001	0.135	0.05	0.32	<0.0001
**DBP**	**Female**	0.549	0.43	0.69	<0.0001	0.577	0.45	0.73	<0.0001
	**Male**	0.17	0.08	0.35	<0.0001	0.175	0.09	0.35	<0.0001
**DTM**	**Female**	0.617	0.47	0.81	0.0004	0.677	0.51	0.89	0.0056
	**Male**	0.163	0.08	0.33	<0.0001	0.189	0.09	0.39	<0.0001
**DCM**	**Female**	0.486	0.36	0.65	<0.0001	0.5	0.37	0.68	<0.0001
	**Male**	0.2	0.11	0.39	<0.0001	0.21	0.11	0.4	<0.0001
**SLR**	**Female**	0.315	0.23	0.43	<0.0001	0.308	0.23	0.42	<0.0001
	**Male**	0.122	0.06	0.26	<0.0001	0.125	0.06	0.26	<0.0001
**KFS**	**Female**	0.075	0.04	0.15	<0.0001	0.076	0.04	0.16	<0.0001
	**Male**	0.104	0.04	0.24	<0.0001	0.109	0.05	0.25	<0.0001
**KES**	**Female**	0.106	0.06	0.19	<0.0001	0.108	0.06	0.19	<0.0001
	**Male**	0.17	0.09	0.32	<0.0001	0.178	0.09	0.34	<0.0001

RR: risk ratio; KGB: knee gap between the point of short head of the biceps femoris at the lateral knee and the surface of the bed; DAP: diameter of muscles connected with the knee joints 4 cm above the patella; DBP: diameter of muscles connected with the knee joints 4 cm below the patella; DTM: diameter of muscles at the thighs; DCM: diameter of muscles at the calves; SLR: straight legs raising in supine; KFS: angle of knee flexion in supine; KES: angle of knee extension in supine; CI: confidence interval.

**Table 6:** Age group wise analyses of Correlation coefficient of 108 experimental patients, female (n=72), male (n=36) between week 0 and week 12

**Parameter**	**Correlation Coefficient between week 0 and week 12**	**Age group: 45-55 years**				**Age group: 55-65 years**				**Age group: 65-75 years**			
		**Right leg**		**Left leg**		**Right leg**		**Left leg**		**Right leg**		**Left leg**	
		**Female (n= 6)**	**Male (n=4)**	**Female (n= 6)**	**Male (n=4)**	**Female (n=16)**	**Male (n=10)**	**Female (n=16)**	**Male (n=10)**	**Female (n=50)**	**Male (n=22)**	**Female (n=50)**	**Male (n=22)**
**KGB**	**Correlation Coefficient**	0.431	0.148	0.229	0.289	0.368	0.71	0.447	0.621	0.314	0.238	0.158	0.341
	**p-value**	0.185	0.812	0.498	0.638	0.045	0.006	0.013	0.023	0.016	0.162	0.235	0.042
**DAP**	**Correlation Coefficient**	0.986	0.527	0.937	0.432	0.980	0.887	0.976	0.838	0.914	0.809	0.904	0.799
	**p-value**	0.000	0.361	0.000	0.467	0.000	0.000	0.000	0.000	0.000	0.000	0.000	0.000
**DBP**	**Correlation Coefficient**	0.646	0.189	0.965	0.487	0.987	0.736	0.991	0.399	0.985	0.892	0.988	0.867
	**p-value**	0.032	0.760	0.000	0.406	0.000	0.004	0.000	0.018	0.000	0.000	0.000	0.000
**DTM**	**Correlation Coefficient**	0.894	0.632	0.963	0.632	0.963	0.010	0.966	0.318	0.908	0.455	0.891	0.470
	**p-value**	0.000	0.367	0.000	0.367	0.000	0.974	0.000	0.290	0.000	0.005	0.000	0.004
**DCM**	**Correlation Coefficient**	0.984	0.510	0.943	0.817	0.836	0.891	0.745	0.724	0.592	0.841	0.485	0.837
	**p-value**	0.000	0.500	0.001	0.184	0.000	0.001	0.000	0.005	0.000	0.000	0.000	0.000
**SLR**	**Correlation Coefficient**	0.108	0.300	0.053	0.400	-0.290	-0.046	-0.275	-0.010	0.324	0.132	-0.185	0.251
	**p-value**	0.752	0.624	0.877	0.505	0.120	0.881	0.141	0.973	0.013	0.444	0.165	0.139
**KFS**	**Correlation Coefficient**	0.692	0.354	0.714	0.341	0.166	0.346	0.194	0.316	0.080	0.368	-0.118	0.273
	**p-value**	0.018	0.559	0.013	0.567	0.380	0.247	0.305	0.292	0.549	0.027	0.378	0.108
**KES**	**Correlation Coefficient**	0.277	0.707	0.295	0.725	0.266	0.566	-0.245	0.392	0.086	0.231	0.155	0.139
	**p-value**	0.409	0.182	0.378	0.165	0.155	0.044	0.192	0.185	0.517	0.175	0.244	0.420

KGB: knee gap between the point of short head of the biceps femoris at the lateral knee and the surface of the bed; DAP: diameter of muscles connected with the knee joints 4 cm above the patella; DBP: diameter of muscles connected with the knee joints 4 cm below the patella; DTM: diameter of muscles at the thighs; DCM: diameter of muscles at the calves; SLR: straight legs raising in supine; KFS: angle of knee flexion in supine; KES: angle of knee extension in supine

**Table 7:** Kellgren-Lawrence (KL) grading scale for knee-osteoarthritis of 108 experimental subjects and 72 Control subjects

**Knee** **joints**	**Gradation**	**Control Group**				**Experimental Group**			
		**Baseline**		**After 12-week**		**Baseline**		**After 12-week**	
		**Number**	**%**	**Number**	**%**	**Number**	**%**	**Number**	**%**
**KOA (Rt. knee)**	Grade-0	None	None	None	None	None	None	None	None
	Grade-1	None	None	None	None	None	None	19	17.59
	Grade-2	None	None	None	None	None	None	42	38.89
	Grade-3	23	31.94	11	15.28	42	38.89	32	29.63
	Grade-4	49	68.05	61	84.72	66	61.11	15	13.89
**KOA (Lt. knee)**	Grade-0	None	None	None	None	None	None	None	None
	Grade-1	None	None	None	None	None	None	24	22.22
	Grade-2	None	None	None	None	None	None	55	50.93
	Grade-3	19	26.39	9	12.50	34	31.48	29	26.85
	Grade-4	53	73.61	63	87.50	74	68.52	None	None

KOA: knee-osteoarthritis

**Figure 4:**
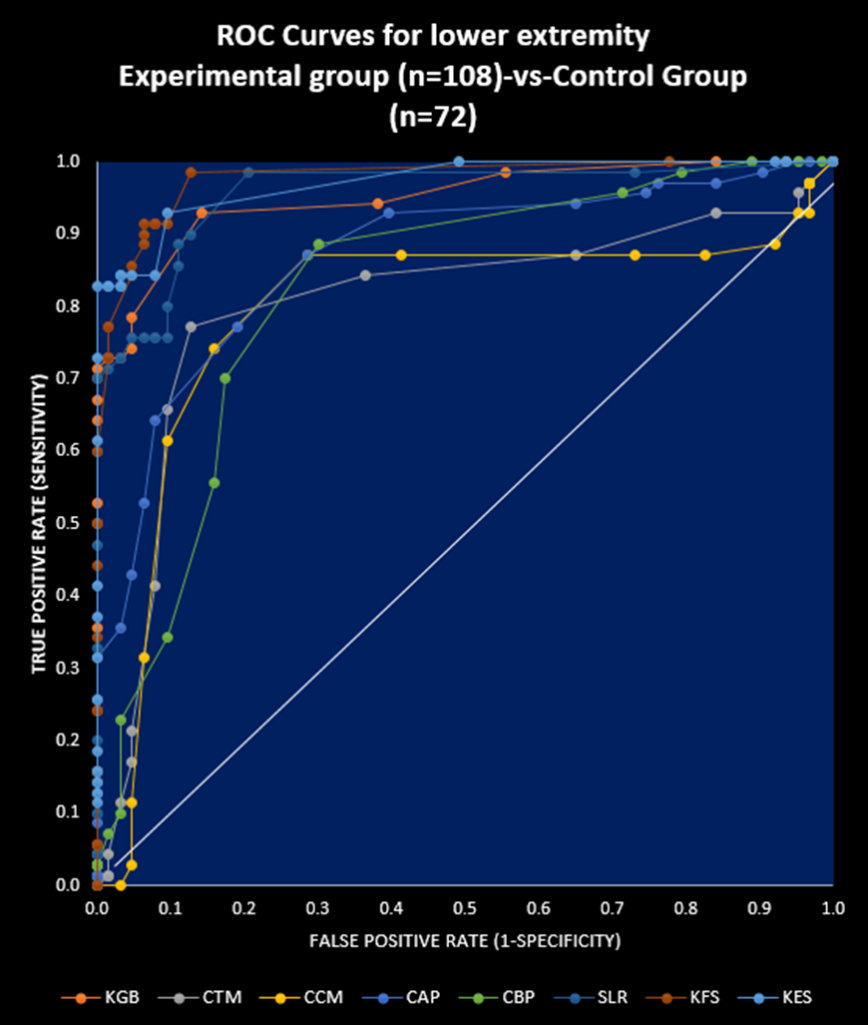
ROC Curves for lower extremity Experimental group (n=108)-vs-Control Group (n=72)

**Pain, stiffness, performance parameters: **The mean levels of improvements: on pain under VAS; on pain, stiffness and physical functions under WOMAC index; of the five separately scored subscales under KOOS knee survey and reduction of body weight confirmed by BMI at week 12 of the treatment with JNS for female-only and male-only patients in the experimental group were all highly significant (p<0.05) compared to the treatment with SYSADOA of female-only and male-only subjects in control group and depicted in figures 6-7. The mean levels of deterioration after 12-week of control group for female-only and male-only patients was not at all significant (data not shown).

**Analysis of the interrater reliabilities and the degree of accuracy of data: **The favorable agreements between the interrater reliabilities and the degree of accuracy for all the anatomical parameters of patients treated with JNS compare to control subjects according to the Cohen’s Kappa (*k*) values were assessed and shown in table 8. 

**Safety and cost evaluation: **The topics of safety and tolerability were evaluated. There were no problems with the supplement and no significant safety problems requiring the suspension of the supplement or altering compliance to the supplementation plan were observed. The use of other drugs including physiotherapy, painkillers and rescue medication also decreased in the JNS group compared to control subjects (*p*<0.05). 

The blood tests relating to liver and kidney function tests such as albumin, bilirubin, alanine aminotransferase (ALT), aspartate aminotransferase (AST), gamma-glutamyl transferase (GGT), alkaline phosphatase (ALP), lactic dehydrogenase (LDH), blood urea nitrogen (BUN) and creatinine were made for the patients treated with JNS before and after the treatment at week-twelve. All the results were shown within normal limits (the analyses of data are not shown).

The average management cost was evaluated for the subjects using JNS. This cost was defined as an average 100% (for the 12 weeks of management) including treatment, diagnostic and loss of working days and these were reduced to 95% with range 79-96 (*p*<0.05).

**Table 8:** Analysis of kappa values of anatomical parameters at twelve-week of 108 experimental patients, female (*n*=72), and male (*n*=36) compare to 72 control patients

**Parameters**	**RIGHT LEG**					**LEFT LEG**				
	**Kappa (k)**	**SE of k**	**95% CI**		**Agreement**	**Kappa (k)**	**SE of k**	**95% CI**		**Agreement**
			**Low**	**High**				**Low**	**High**	
**KGB**	0.74	0.05	0.644	0.839	Substantial	0.73	0.05	0.634	0.83	Substantial
**DAP**	0.38	0.069	0.241	0.512	Fair	0.41	0.068	0.278	0.545	Moderate
**DBP**	0.45	0.064	0.327	0.577	Moderate	0.46	0.062	0.334	0.579	Moderate
**DTM**	0.34	0.068	0.202	0.47	Fair	0.33	0.068	0.197	0.462	Fair
**DCM**	0.45	0.067	0.316	0.578	Moderate	0.42	0.066	0.292	0.552	Moderate
**SLR**	0.67	0.054	0.562	0.774	Substantial	0.65	0.054	0.543	0.756	Substantial
**KFS**	0.84	0.041	0.759	0.92	Almost perfect	0.81	0.045	0.718	0.893	Almost perfect
**KES**	0.72	0.051	0.62	0.821	Substantial	0.72	0.051	0.62	0.821	Substantial

Agreement interpretation of k value: <0: Poor; 0.00-0.20: Slight; 0.21-0.40: Fair; 0.41 - 0.60: Moderate; 0.61 - 0.80: Substantial; 0.81 - 1.00: Almost perfect.

**Figure 5:**
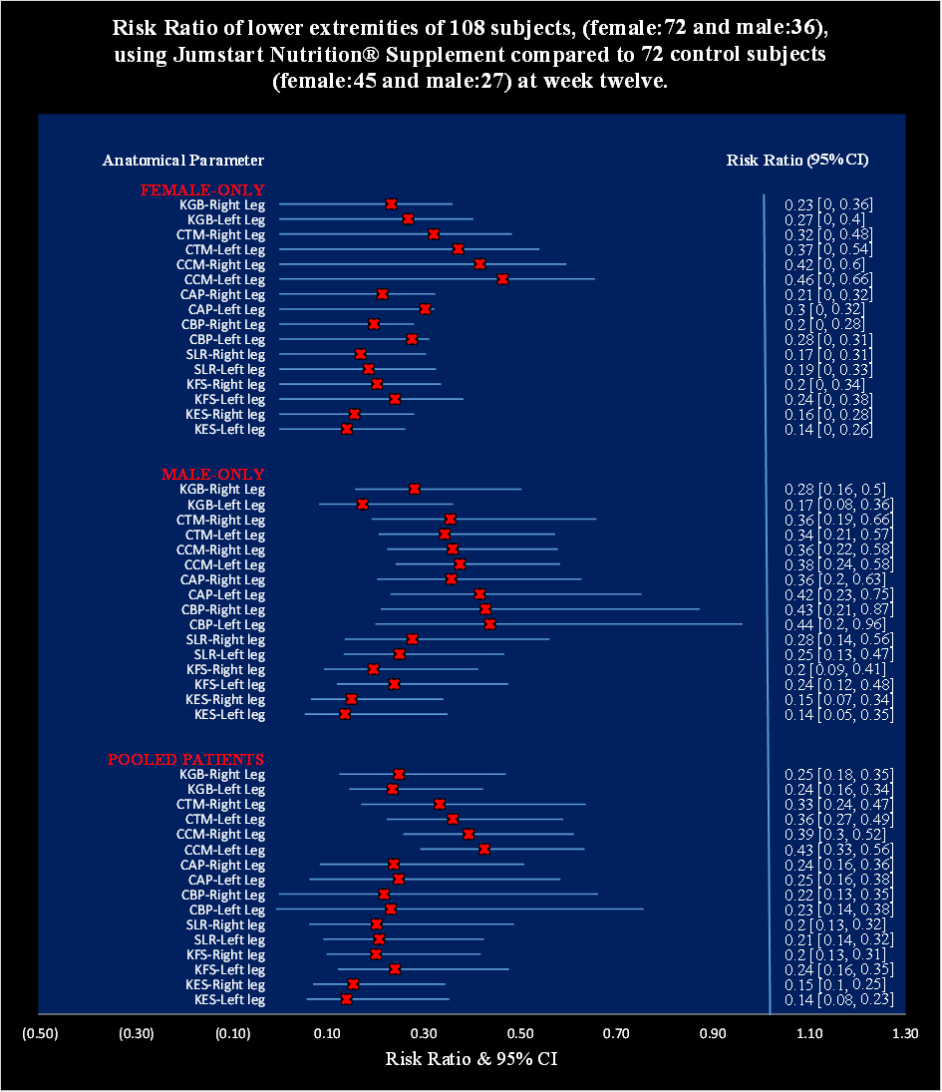
Risk Ratio of lower extremities of 108 subjects, (female: 72 and male: 36), using Jumpstart Nutrition® Supplement compared to 72 control subjects (female: 45 and male: 27) at week twelve.

(RR: risk ratio; KGB: knee gap between the point of short head of the biceps femoris at the lateral knee and the surface of the bed; DAP: diameter of muscles connected with the knee joints 4 cm above the patella; DBP: diameter of muscles connected with the knee joints 4 cm below the patella; DTM: diameter of muscles at the thighs; DCM: diameter of muscles at the calves; SLR: straight legs raising in supine; KFS: angle of knee flexion in supine; KES: angle of knee extension in supine; CI: confidence interval)

**Figure 6:**
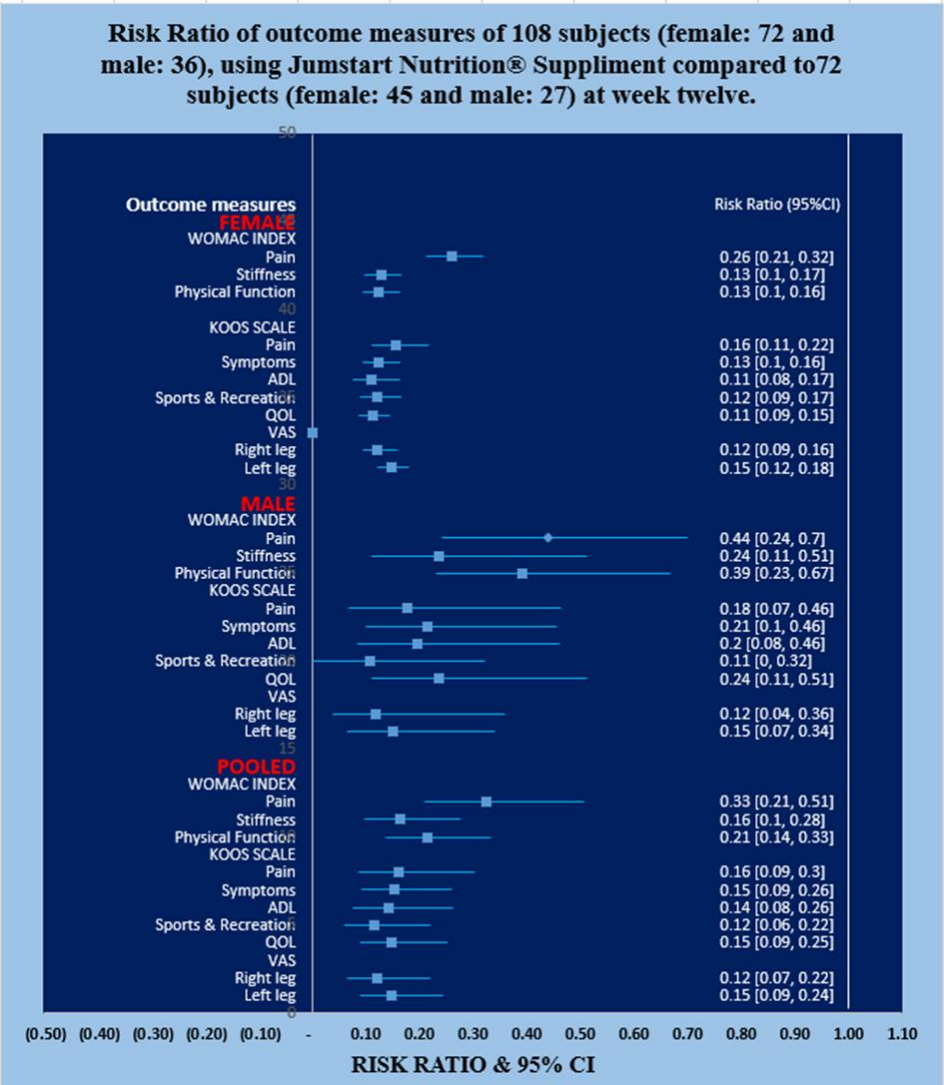
Risk Ratio of outcome measures of 108 subjects, (female: 72 and male: 36), using Jumstart Nutrition® Supplement compared to 72 control subjects (female: 45 and male:27) at week twelve

**Figure 7:**
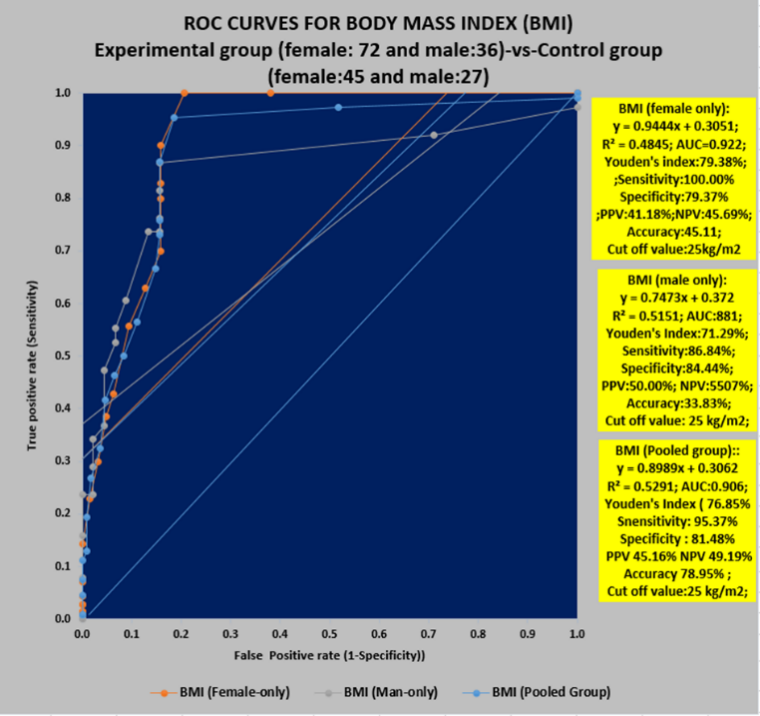
ROC curves for body mass index (BMI) Experimental group (female: 72 and male: 36) vs Control group (female: 45 and male: 27(

**Figure 8:**
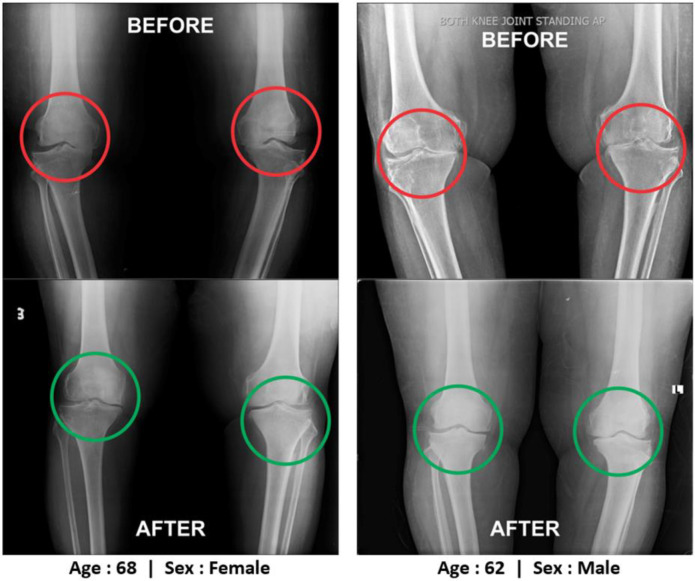
Radiological images of the patients with OA before and after the treatment with Jumpstart Nutrition® supplement at week 12

## Discussion

To our knowledge, this is the first study to investigate the effects of aberrant lower extremity and outcome measures on OA patients with the treatment of JNS compared to the treatment with SYSADOA. We found that the women with OA changes are more prevailing than men with the age group 65-75 years in experimental group than control group which proclaim the prediction made by WHO so far as the gender as well as age-limit of people are concerned (3-4).

The present results suggest that there is a close relationship of the risk factors between the asymmetrical lower extremity (KGB, DAP, DBP, DCM, DTM, SLR, KFS, and KES) (tables 2A-6) and the pain with disable-related outcome measures including obesity (VAS, WOMAC index, KOOS, and BMI) (figures 5-6) in OA.

There are no medications available to cure OA permanently except some kinds of non-steroidal anti-inflammatory drugs (NSAIDs) including SYSADOA, corticosteroids for relieving pain, inflammation, stiffness, improvement of quality of life temporarily. But they have tremendous side-effects like cardiovascular and gastrointestinal problems (14). 

Transgressed with the conventional treatment protocol, the new evidence could potentially increase public interest in the benefits of alternative treatments. According to Basedown et al., currently 69% of patients with OA take some form of dietary supplements for their condition as an alternative therapy (5). 

According to Lang (36) muscles support the bone structure, particularly during the process of growth and development. The close relationship between muscle and bone are observed through the mechanostat theory, evaluated firstly to determine gender differences by Frost (37-38). During the attainment of aging, peak bone and muscle strength, both men and women begin to lose both bone and muscle tissues and possibilities of muscles and bone damage widely known as sarcopenia (39-42). 

Interestingly, researchers have investigated knee-osteoarthritis (KOA), which occurs due to the damage of leg muscles such as quadriceps femoris, extensor muscle, etc. The quadriceps strength (peak torque generation) is an important evaluation for physical function in men and women (43-47) for the evolution of weakness of the quadriceps muscle during OA. The bones, muscles and cartilage are the important tissues deformed during OA as an evidenced by radiographic marker (48-49). The author has further established that the deranged lower anatomical features are the risk factors for OA (50) and established the normalization of the deranged anatomical features for OA with the help of established phytotherapy (51-59). 

The present results indicate that various anatomical parameters are deranged, may be due to muscle wasting, weakness and degeneration, connective tissue damage, potential loss of joint cartilage, bone hypertrophy and joint effusion as a result of which both the legs become asymmetrical, when patient suffers with OA. 

When we further analyzed the limb-wise deformities, it was observed that the KGBs increased and not touching the back of knee joints (popliteal region) on the bed while supine. They become asymmetrical may be due to the cumulative effects of muscle wasting, weakness, inflammation and stiffness of the connective muscles during the disease condition. This further increased may be due to the prolonged use of knee supports, hyaluronic acid injections or corticosteroid injections or arthrocentesis are used for quick diminishing of pain, stiffness, inflammation and for increasing the strength of muscles temporally.

Our results show that there is reduction of KGB by 59.98% and 61.45% in the right and left legs for female-only and by 53.53% and 55.43% in the right and left legs for man-only respectively by using JNS in the experimental pooled group (45-75 years) and they become symmetry at week-12 compared to week 0 (table 2A-B), where as in the control pooled group (45-75 years), these were decreased by 3.56% and 2.31% for right and left legs for female-only and by 2.84% and 2.57% in the right and left leg for man-only respectively and they remain asymmetry at week-12 compared to week 0 (table 2A-B).The cumulative effects of muscular wasting, inflammation, effusion or blood clotting due to engorgement of saphenous vein, probably be the reason of the asymmetry of DAP due to the inflammation over the medial tibiofemoral joints. It may be the further reason of using knee braces, hyaluronic acid injections or corticosteroid injections or arthrocentesis for quick diminishing of pain, stiffness and inflammation. Moreover, the radiological images indicate that there is marked effusion at medial tibiofemoral joint due to severe damage of cartilage during OA (Figure 4). At the same time, the cumulative effects of muscular wasting, inflammation, effusion or blood clotting on the anterior, posterior, lateral and medial parts of lower legs especially tibialis and anterior extensor hallucis longus and digitorial longus, gastrocnomius, soleus, and Achilles tendon may be the causing effects of asymmetry for both the DBPs. The radiological imaging of the knee joints confirmed the same.

Our results indicate that there is reduction of DAP and DBP by 6.79% and 4.03% in the right knee joint respectively and that to left knee joints by 7.44% and 6.25% for female-only and by 8.62% and 8.18% in the right knee joint and that to left knee joint by 9.62% and 9.81% for man-only respectively by using JNS in the experimental pooled group (45-75 years) and they become symmetry at week-12 (table 2A-B), where as in the control pooled group (45-75 years), these were diminished by 0.21% and 0.16% in case of DAP and 0.23% and 0.22% in case of DBP for right and left legs respectively for female-only and that of by 0.72% and 0.21% in case of DAP and 0.14% and 0.20% in case of DBP for right and left legs respectively for man-only and they remain asymmetry at week-12 (Table 2A-B). 

It is further observed that there is increased in asymmetrical of DTM between the legs, probably due to the cumulative effects of muscular wasting/ muscular bulging in the posterior region of the thighs and may be commonly compressed the sciatic nerve which is originating from the tubersity of the ischium and inserting to the tibia resulting which patient is complained the acute or mild pain in the lumbar region along with knee pain. The reason for decreased asymmetrical DCM of both legs may be due to cumulative muscular wasting / stiffness of gastrocnemius muscles for which further compressed the tibial nerve which is a branch of sciatic nerve, may be affected of prolong use of knee supports, tenderness of Achilles tendon, soleus, calcaneus spurs, rigidity of ankle joints and such other reasons. Therefore, researchers have identified that the slight difference of the diameter of the calf muscle of two legs trigger up the compression in the lumbar vertebrae as they misaligned (51-53).

The present results show that there is an increase in muscles diameter of DTM by 2.05% and 1.97% in the right and left legs for female-only and by 6.82% and 6.32% in the right and left legs for man-only respectively by using JNS in the experimental pooled group (45-75 years) and they become symmetry at week 12 (table 3A-B), whereas in the control pooled group, those who are using SYSADOA, these were improved by 0.10% and 0.10% for right and left legs for female-only and by 0.04% and 0.06% in the right and left leg for man-only respectively and they remain asymmetry at week-12 (table 3A-B). Furthermore, the present results show that DCM has increased by 1.22% and 1.16% in the right and left legs for female-only and that increased by 2.86% and 2.26% in the right and left legs for male-only and they become symmetry at week-12 with JNS in the experimental pooled group (45-75 years (table 3A-B) but there were no improvements in the muscle diameter of the right and left legs for female-only and that for male-only and remain as asymmetrical at week-12 in the control pooled group (45-75 years) when used SYSADOA (table 3A-B) 

It is well known that the knee flexion and extension are two main activities of the movement of the knee joint. All the muscles that move these joints are in the anterior, posterior and lateral thigh region and for the flexion activity the muscles nerve root is sciatic nerve and for extension is femoral nerve. It reveals from the present results that SLR, and KFS are all reduced and at the same time the KES of both legs increased during OA, the reasons for the same may be due to massive muscular wasting, stiffness and muscle degeneration especially the quadriceps muscles and tendons and healthy muscle fibers replaced by fibrosis and fat making muscle tissues in the thigh region and the legs probably the reason of asymmetry between the legs for SLR, KFS, and KES. 

The results indicate that there is an increase of angles of SLR and KFS by 91.60% and 27.89% in the right leg and that to left leg by 95.10% and 27.69% for female-only and by 60.54% and 24.68% in the right leg and that to left leg by 62.65% and 23.97% for man-only respectively and they become symmetry at week 12 by using JNS in the experimental pooled group (45-75) (table 4A-B), whereas in the control pooled group, these were further reduced by 0.29% and 0.31% in case of SLR and 0.13% and 0.12% in case of KFS for right and left legs respectively for female-only and that of by 0.22% and 0.23% in case of SLR and 0.16% and 0.12% in case of KFS for right and left legs respectively for man-only and they remain asymmetry at week-12 (table 4A-B). Furthermore, in the experimental pooled group, there is diminution of angles of KES by 51.52% and 51.57% in the right and left legs for female-only and the same has be reduced by 45.23% and 44.38% in the right and left legs for man-only and they become symmetry at week-12 with JNS (table 4) whereas patients under control pooled group (45-75), the increased levels of KES angle were recorded as 0.75% and 0.56% in the right and left legs for female-only and the same was recorded as 0.62% and 0.70% in the right and left leg respectively for man-only and they remain as asymmetrical at week12 (table 4A-B )

Finally, table 5 shows the risk ratios of all the parameters of lower extremity of OA patients treated with JNS at week 12 were highly significant (p<0.0001) compared to control group treated with SYSADOA.

Therefore, in the present supplementary study, the vitamin CoQ_10_ and vitamin K_2_ are mixed because of CoQ_10_ levels decrease with aging, and this loss of antioxidant power coupled with increased reactive oxygen species (ROS) production can result in an age-related oxidative stress that can influence the development of other metabolic conditions (60) and vitamin K_2_ in the form of MK-7 (menaquinone form , where 7 signifies the number of 5-carbon units), has been shown to be a bioactive compound in regulating osteoporosis, atherosclerosis, cancer and inflammatory diseases without risk of negative side effects or overdosing (61). 

Pain syndromes, inflammation and impaired quality of life are the major perception factors among patients in any musculoskeletal disorders especially OA (15, 62-63). To overcome these phenomenon, boswellic acids and curcumin are added with this supplement (64). The author has already been elaborated in detail the significance of the ingredients (boswellic acids and curcumin) and cost-effectiveness used in the supplement in the previous article (15, 20). This powerful antioxidant (64) is now available in a new delivery system (Jumpstart Nutrition®, Nanophyto Wellness Private Ltd, Kolkata, India) that improves the bioavailability of curcuminoids and boswellic acids (15, 19, 20).

In the present study, it indicates that all the internationally-acclaimed pain-related parameters under VAS, WOMAC index, and KOOS are in much favorable positions (decreasing levels of pain activities and increasing levels of lifestyle) of the patients under experimental group at week 12 treatment protocol (figures 5-6) compared to control group Therefore, JNS exerts a significant anti-inflammatory action in these patients, leading to a reduction in symptoms of pain and disability and the compositions can be used as supplement to achieve the symmetrical effects of lower extremity in OA patients. 

Again, obesity is the important phenomenon for developing OA as reduced quadriceps strength relative to body weight is a risk factor for knee OA (65). Therefore, vitamin-K_2_ is the important ingredient in the supplement. Several researchers have shown that vitamin-K_2_ intake may support reducing body weight, waist circumference, body composition, visceral fat (66-68). Moreover, the vitamin-K_2_ treatment supports osteogenic differentiation within bone marrow mesenchymal stem cells (69, 70).

Figure 6 shows that there is substantial reduction of bodyweight due to supplementary diet mixed with vitamin-K_2_. In the present study, the reduction of bodyweights is recorded as 13.13 % for female-only and 11.04% for man-only in the experimental group compared to control.

Our results show the probable improvements on reducing osteophytes, joint space narrowing, sclerosis, bony deformities of knee-joints of the OA patients treated with JNS under experimental group as assessed by K-L grading scale. More numbers of patients of supplementary group have shifted to higher grades (grade 2 and grade 3) from grade 4 for both right and left knee joints with JNS as against further deterioration of OA in the control group (table 6 and figure 4). The author has further elaborated in detail the functions of calcium, phosphorus, vitamin-K_2_, vitamin-D_2_, coenzyme-Q_10_, soy and whey proteins contain in JNS for the improvement of OA patients in the previous study (15).

Finally, it is to be observed that there are almost perfect to fair agreement between the interrater reliabilities and the degree of accuracy for all the anatomical parameters of patients treated with JNS compared to control subjects according to the Cohen’s Kappa (*k*) valuations (table 8). 

However, this study has several important limitations. Firstly, the biasedness of the results has not been checked as we have taken a small sample size. The supplement was studied at short term (≤3 months) but we are unsure whether equivalent efficacy and safety would have been achieved in the long term. Secondly, the present study relied predominantly on x-ray based determination of OA, but higher resolution magnetic resonance imaging (MRI) modalities may prove to be a stronger clinical correlation than symptoms and radiographic measures with the more sensitive measure of cartilage pathology. Because research showed that meniscal subluxation is a risk factor for cartilage loss and joints space narrowing in people with symptomatic KOA (71, 72) and conflicting evidence is found for the association between meniscal subluxation and pain (73). Thirdly, patients restricted to be treated with supplement are those suffering from the following disorders: adverse pathogenic effects on milk products; concomitant diseases required parallel multiple drug treatment; a history of cancer including caranomatosis and granulocytic leukemia; a history of chronic liver, heart and kidney diseases; patients refuse to do x-rays, a physical evaluation and /or attend weekly follow-up visits; patients with dementia, morbid obesity, pregnancy, prior knee surgery. In addition to that patients with amputated legs are restricted to this study. 

In conclusion, this supplement registry study suggests that Jumps Jumpstart Nutrition® can be considered as an effective bone, and muscle nutrition supplementary management of OA patients for the improvement of risk factors by achieving the symmetries in the deranged lower anatomical parameters such as KGB, DAP, DBP, DTM, DCM, SLR, KFS, and KES (tables 2A-6) confirming findings with knee joint radiographic images correlated with Kellgren-Lawrence grading scale (table 7 and figure 4) and improving the internationally approved outcome measures along with functional activities including bodyweight under VAS, WOMAC, KOOS, and BMI (figures 6-7). 

Further research is suggested to be under taken for the evaluation of inflammation, muscular dystrophy, connective tissue damage and skeletal muscles damage with the help of analyses of suitable biomarkers such as interleukin-10, tumor necrosis factor-alpha, C-reactive protein, creatine kinase-muscle and aldolase-A by using Jumpstart Nutrition® supplement in other musculoskeletal diseases such as rheumatoid arthritis.
